# Complement receptor *C3ar1* deficiency does not alter brain structure or functional connectivity across early life development

**DOI:** 10.1093/braincomms/fcaf422

**Published:** 2025-10-28

**Authors:** Hanna Lemmik, Eugene Kim, Eilidh MacNicol, Davide Maselli, Michel Bernanos, Zhuoni Li, Dauda Abdullahi, Esther Walters, Maria Elisa Serrano Navacerrada, Wuding Zhou, Aleksandar Ivetic, Diana Cash, Laura Westacott

**Affiliations:** Department of Neuroimaging, King’s College London, London SE5 8AF, UK; Department of Neuroimaging, King’s College London, London SE5 8AF, UK; Department of Neuroimaging, King’s College London, London SE5 8AF, UK; School of Cardiovascular and Metabolic Medicine & Sciences, King’s College London, London SE5 9NU, UK; Department of Neuroimaging, King’s College London, London SE5 8AF, UK; Department of Forensic and Neurodevelopmental Sciences, King’s College London, London SE5 8AF, UK; Department of Neuroimaging, King’s College London, London SE5 8AF, UK; Department of Human Anatomy, College of Medical Sciences, Abubakar Tafawa Balewa University, Bauchi 8R68+VX, Nigeria; Department of Neuroimaging, King’s College London, London SE5 8AF, UK; Department of Neuroimaging, King’s College London, London SE5 8AF, UK; Peter Gorer Department of Immunobiology, King’s College London, London SE1 9RT, UK; School of Cardiovascular and Metabolic Medicine & Sciences, King’s College London, London SE5 9NU, UK; Department of Neuroimaging, King’s College London, London SE5 8AF, UK; Neuroscience and Mental Health Innovation Institute, Cardiff University, Cardiff CF24 4HQ, UK

**Keywords:** C3aR1, behaviour, tensor-based morphometry, fractional anisotropy, BOLD fMRI

## Abstract

Genetic deletion of the complement C3a anaphylatoxin chemotactic receptor (*C3ar1*), a key component of the innate immune response, is reported to induce behavioural phenotypes resembling anxiety and hyperactivity in mice, suggesting a neurodevelopmental role for this gene in health. However, it is not currently clear when and where *C3ar1* is needed in the brain, which is further complicated by the fact that *C3ar1* is expressed predominantly by microglia and therefore does not localize to specific brain regions, warranting exploratory and brain-wide assessment through neuroimaging. Resolving when and where *C3ar1* is needed are questions of significant translational importance because, as a G-protein-coupled receptor, human C3AR1 serves as a potential therapeutic target for disorders associated with complement upregulation, such as schizophrenia. To provide a brain-wide assessment of developmental *C3ar1* activity, we used longitudinal MRI in male and female adolescent and adult mice (*N* = 34 *C3ar1^tm1Cge/tm1Cge^* and *N* = 35 *C3ar1^+/+^*) to estimate regional brain volume using tensor based morphometry, white matter microstructure using fractional anisotropy from diffusion-weighted MRI, and functional connectivity from blood oxygen-level dependent MRI, with behavioural assessment in adulthood. We repeated structural MRI measures in this cohort *ex vivo* to achieve higher resolution. We further repeated *in vivo* structural assessment preceded by behavioural testing in adulthood in a second cohort of mice (*N*  *=* 20 *C3ar1^tm1Cge/tm1Cge^* and *N*  *=* 19 *C3ar1^+/+^*) to improve confidence in our findings. We achieved low regional brain volume variability, allowing us to resolve previously reported sexually dimorphic effects. We were further able to confirm a well-known developmental increase in fractional anisotropy. Despite being able to detect these established effects, we did not find a robust *C3ar1*-dependent phenotype in any of the measures we tested, including behaviour, which may be attributed to our study being the first behavioural study in *C3ar1*-deficient mice to include littermate controls. Therefore, our data do not support neurodevelopmental hypotheses for *C3ar1*, which is encouraging for therapeutic strategies targeting this receptor since interventions are unlikely to disrupt brain development.

## Teaser

Lemmik *et al.* report that genetic deletion of *C3ar1* in mice does not result in behavioural changes nor brain changes as measured by structural and functional MRI, despite capturing well known sexually dimorphic and developmental effects in their datasets. This finding supports the safety of interventions aimed at this receptor.

## Graphical Abstract

**Graphical Abstract:**
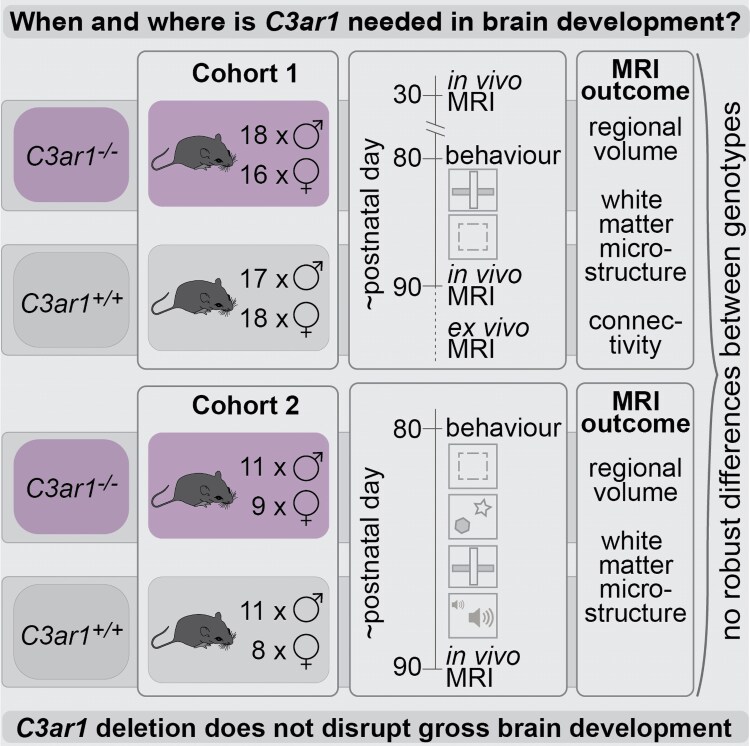

## Introduction

The complement system is a conserved immune pathway that participates in host defence through pathogen clearance and regulating inflammation^1^ as well as tissue homeostasis,^2,3^ with emerging roles in neurodevelopment, psychiatric disorders and neurodegeneration.^4-6^

The most convincing evidence for the involvement of the complement system in neurodevelopment so far is its genetic association with schizophrenia. Schizophrenia is a complex and highly heritable neurodevelopmental disorder characterized by hallucinations, delusions, and impaired cognition, with symptoms typically emerging in late adolescence or early adulthood.^7^ Neurobiological hallmarks of schizophrenia include grey matter loss^8^ and a reduction in synaptic density.^9^ Genome-wide association studies (GWAS) of schizophrenia have helped to identify two complement-related risk loci; a structural variant in the complement component 4 A (*C4A*) gene,^6^ which encodes the C4A protein responsible for propagation of complement activation, and a variant in the CUB and Sushi Multiple Domains 1 (*CMSD1*) gene, which encodes a putative complement inhibitor protein.^10,11^ Preclinical studies link these variants to increased brain-specific complement activation and synapse loss,^6,11,12^ potentially tying complement activation to synaptic pathology in schizophrenia. Indeed, the *C4A* schizophrenia-risk genotype associates with MRI markers of grey matter loss and reduced cognitive performance in humans, even in the absence of neurological disorders.^13^ Further, patients with elevated complement proteins have more severe negative symptoms in psychosis,^14^ which typically do not respond to anti-psychotic medication. Complement modulation therefore has potential in addressing this unmet therapeutic need.

C3a anaphylatoxin chemotactic receptor (C3aR1), a G-protein coupled receptor (GPCR) bound by complement activation product C3a and the granin family neuropeptide TLQP-21,^15^ acts downstream of complement activation and stands out as a pharmacologically tractable target for modifying complement activity in the brain.^16^ In the brain, *C3ar1* transcripts are predominantly expressed by microglia according to Allen Brain Map Transcriptomic explorer mouse and human data (microglia markers *Itgam*, *Cx3cr1* and *C1qb*) and Stevens lab microglia single cell atlas (sorted by FACS; *Cd45^low^*, *Cd11b^high^*, *Cx3cr1^high^*), with minimal neuronal expression.^17,18^ While its temporal expression patterns are not yet well characterized, C3aR1 appears to be active during early embryonic development, potentially influencing progenitor cell proliferation.^17,19,20^ C3aR1 also appears to facilitate developmental astrocyte phagocytosis by microglia in the retina,^21^ as well as to regulate microglial reactivity and neuroinflammation more broadly.^22-27^ Brain morphological changes observed in *C3ar1*-deficient mice further support its neurodevelopmental relevance,^28,29^ although there is no consensus on the precise neurodevelopmental actions of C3aR1. Addressing this gap is important given this receptor’s potential as a pharmacological target.

C3aR1 signalling may impact brain functions relevant to psychiatric symptomatology since a range of behavioural phenotypes have been reported in *C3ar1*-deficienct mice. These include abnormal anxiety-like behaviours,^30^ hyperactivity,^29^ cognitive impairment^19^ but also a resilience to depressive-like behaviours induced by chronic stress or inflammation.^31-33^ Although the observed involvement of C3aR1 in adult behaviour suggests that it is needed for normal brain function, previous studies have not addressed the question of whether these phenotypes arise because of a C3aR1 deficit during development or because it is continuously needed, which can be resolved through longitudinal assessment. Another important yet overlooked aspect is the use of the appropriate wild-type *littermate* control animals that none of the aforementioned studies included. Instead, these studies used separately raised cohorts of control and mutant mice which represents a known confound due to the rapidly diverging genetic backgrounds in small inbred colonies,^34^ but also because litter environment affects behaviour and brain development.^35-37^

To investigate the potential consequences of *C3ar1* deficiency during development, we adopted a global, unbiased approach, conducting a longitudinal study of male and female *C3ar1*-deficient mice and their wild-type littermates during adolescence (postnatal day, PND27–31) and adulthood (PND81–92). Using structural and diffusion magnetic resonance imaging (MRI and dMRI), as well as resting state fMRI (rsfMRI), we aimed to assess whether the absence of C3aR1 affects brain development during adolescence—a critical period for psychiatric vulnerability^38,39^—or whether this requirement only becomes evident by adulthood.

Our imaging measures included regional brain volumes derived from tensor-based morphometry (TBM), fractional anisotropy from dMRI to evaluate white matter organization, which is influenced by microglial activity during development,^40,41^ and graph theoretical analysis of functional connectivity (FC) correlates of blood oxygenation level dependent (BOLD) signal, such as global efficiency and clustering coefficient to characterize brain network topology.^42-44^ These techniques were complemented by behavioural testing in adult mice that measured cognition and emotional reactivity, as we sought to replicate previously reported behavioural experiments in *C3ar1*-deficient mice.^19,29,30^ Unexpectedly, we found no robust brain or behavioural phenotype in our datasets, challenging the previous assumptions of a neurodevelopmental role for C3aR1 under physiological conditions.

## Methods and materials

### Animals

All animal procedures complied with the UK Animals and Scientific Procedures Act 1986 and were approved by the local ethical committee at King’s College London (KCL). Homozygous *C3ar1^tm1Cge/tm1Cge^* mice were generated by homologous recombination in embryonic stem cells and kindly provided by Dr. Bao Lu and Prof. Craig Gerard (Harvard Medical School, Boston, MA).^45^ These mice were subsequently backcrossed onto the C57BL/6J strain for at least 12 generations and maintained on a C57BL/6J background in Professor Wuding Zhou’s laboratory in KCL. For this study, cryopreserved stocks were rederived in KCL and crossed to C57BL/6J mice purchased from Charles River to refresh the genetic background following Jackson’s Laboratories line refreshing protocol. **See Supplemental methods for further notes on experimental animals**.

### Validation of mutation

Bone marrow-derived macrophages were obtained from tibias and femurs of eight three-month-old mice (*N =* 4 *C3ar1^+/+^, N =* 4 *C3ar1^tm1Cge/tm1Cge^*). Bone marrow suspension was filtered through a 40 µm mesh, centrifuged at 450 x g for 5 min at 4°C, and treated with NH_4_Cl haemolysis buffer (NH_4_Cl 0.15 M, KHCO_3_ 0.01 M, EDTA 0.0001 M). After a second centrifugation under the same conditions, cells were washed with PBS and resuspended in Gibco RPMI 1640 Medium (Thermo Fisher, #21875-034) supplemented with 50 ng/ml recombinant mouse macrophage colony-stimulating factor (M-CSF, R&D Systems, #416-ML-010/CF), 1% penicillin, 1% streptomycin, and 10% heat-inactivated foetal bovine serum (Sigma, #F9665-50 ml).

Cells were seeded at 1 × 10^6^ cells/ml in six-well plates (six wells per animal) and incubated at 37°C with 5% CO_2_ for 72 h. On day three, the medium was refreshed, and 50 ng/ml recombinant mouse IL4 (R&D Systems, #404-ML-010/CF) was added to half of the wells to skew them towards M2 phenotype. Incubation continued for an additional 48–72 h, depending on cell confluence.

RNA extraction, cDNA synthesis, gel electrophoresis and qPCR were conducted using standard protocols, details of which can be found in the Supplementary methods.

### Study design

This study used two separate cohorts of male and female *C3ar1*-deficient *C3ar1^tm1Cge/tm1Cge^* and littermate wild-type mice. The main, longitudinal MRI cohort (*N =* 35 *C3ar1^+/+^, N =* 34 *C3ar1^tm1Cge/tm1Cge^*, *N* = 20 litters), termed Cohort 1, had *in vivo* MRI performed in adolescence (range 27–31 days) and adulthood (range 81–92 days), and the adulthood MRI scan was preceded by the open field (OF) and elevated plus maze (EPM) tests. The adolescence time-point was chosen because mice reach puberty approximately between PND24–34.^46-48^ Synaptic remodelling occurs in two waves in mice and humans—during early perinatal development and in adolescence.^38^ By including a pre-pubertal timepoint, we aimed to determine whether changes occur during the initial wave of pruning or emerge specifically between adolescence and adulthood. Behavioural testing was limited to adult animals, as aberrant behavioural phenotypes in *C3ar1* knockouts have only been reported during adulthood to date. *Ex vivo* imaging was conducted in Cohort 1’s perfusion-fixed brains after the final adulthood scan.

For Cohort 2 (*N =* 19 *C3ar1^+/+^, N =* 20 *C3ar1^tm1Cge/tm1Cge^*, *N* = 13 litters), behavioural testing was conducted similarly to Cohort 1 in adulthood only (range 74–110), and consisted of OF, novel object recognition (NOR), EPM and prepulse inhibition (PPI) tests followed by *in vivo* structural and diffusion MRI (note that MRI was not conducted in adolescence in this cohort). For both cohorts, behavioural testing was conducted 2–7 days before the adulthood scan. **See**  Supplemental methods  **for behavioural experiment details and**  Supplemental Fig. 1  **for genotype and sex ratios of experimental animals**.

The sample size of Cohort 1 was statistically powered to detect medium effect sizes in regional volume using TBM across four groups (males and females analysed separately), with a minimum sample size of *N =* 15 per group based on previously observed variance with this method by our group.^49^ Cohort 2 sample size was powered to detect medium effect sizes in regional volume with sexes combined, *N =* 16 per group.

### In vivo MRI

Two to three days after behavioural testing, mice were imaged using a Bruker BioSpec 9.4 T scanner with an 86-mm volume resonator for transmission and a 4-channel surface array coil. Anaesthesia was induced with 4% isoflurane in medical air (1 L/min) and oxygen (0.4 L/min), maintained at 2% but adjusted based on respiration rates. For BOLD fMRI in Cohort 1, we used a medetomidine and isoflurane anaesthesia optimized for mouse fMRI.^50^ This consisted of a subcutaneous medetomidine bolus (0.05 mg/kg) followed ten minutes later by its continuous infusion (0.1 mg/kg/h), with isoflurane levels gradually reduced to 0.45–0.65% over 15 min from the start of the infusion. BOLD fMRI was conducted after the structural scans, which took a further 45–60 min after reducing isoflurane level. The respiration rate was monitored with a pressure sensor, and temperature was monitored with a rectal thermometer and maintained at 36–37°C using a water circulation system. **For image pre-processing and further MRI details, see**  Supplemental methods.

### Study templates

The antsMultivariateTemplateConstruction2.sh script from ANTs was used to create study specific templates from processed images. For *in vivo* scans of Cohort 1, magnetization transfer weighted (MTw), T1w, R2* map (generated from the multi-gradient-echo proton density weighted, T1w and MTw images using the qi mpm_r2 s command in the QUIT package), S0, fractional anisotropy (FA) and mean diffusivity (MD) images were used. For the *ex vivo* scans of Cohort 1, separate T2w and diffusion weighted images (DWI) templates were created comprising S0 which is an estimated non-diffusion-weighted image from dtifit, FA, and MD images. For Cohort 2, PDw, T1w, MTw, S0, FA, and MD images were used. For BOLD fMRI, the study template was a T2w image derived from a separate mouse study conducted at the BRAIN Centre (KCL). The use of this external template was justified by the low resolution of fMRI, which does not benefit from creating a study-specific template.

### Jacobian determinant maps

To estimate volume, Jacobian determinant maps were generated from the deformation fields corresponding to the transformation of each subject to the study template using the CreateJacobianDeterminantImage command from ANTs. The Jacobian determinant values of all voxels within the template brain mask were summed to obtain the total brain volume of each subject. Jacobian determinants were calculated from the combined rigid, affine, and Symmetric Normalization (SyN) transforms as well as from only the SyN transforms to obtain maps of absolute and relative volume (accounting for differences in global brain volume), respectively. For TBM, the Jacobian determinants were subsequently log-transformed.

### Voxel-wise analysis

For voxel-wise statistics, FSL randomize with permutation testing was used (10 000 for Cohort 1, 5000 iterations for Cohort 2) followed by a threshold-free cluster enhancement (TFCE) and family-wise error (FWE) correction as described previously.^51,52^ Given the absence of genotype differences in total brain volume, TBM regional volumes are reported relative to total brain size for greater accuracy.^53^ For sex differences, absolute volume maps are reported in the Supplemental material.

### Common coordinate space

The study template was registered to the Allen Mouse Brain Common Coordinate Framework^54^ using ANTs, and the Allen atlas was subsequently transformed to the study template space with the inverse transform.

### ROI-based analysis of volume

The images were segmented using an in-house modified version of the Allen atlas consisting of 72 regions.^49,54^ These segmentations were subsequently used to compute regional volumes by summing the Jacobian determinants within each parcellation.

Regional volume variability was estimated by calculating a coefficient of variation (CV) for each region with normalized root-mean-square method for each genotype in each experiment, using the calculation: $CV\;=\;\frac{\sigma }{\mu }$, where *σ* is the standard deviation and *μ* the population mean for each region in the atlas (*N =* 72 regions). Group differences in CV were calculated with a Kruskal-Wallis test (SciPy.stats, kruskal).

### Fractional anisotropy

For mass-univariate voxel-wise analysis of fractional anisotropy, values from dtifit for Cohort 1 were again analysed with FSL randomize with permutation testing (10 000 permutations) followed by TFCE and FWE-correction. Fractional anisotropy is not reported for Cohort 2 in this manuscript for brevity (null results). For ROI-based analysis, voxel FA medians within parcellation were used for all white matter regions.

To calculate the change over time in fractional anisotropy in the longitudinal study, adolescence values for each voxel value or regional median were subtracted from adulthood values. Differences between genotypes were calculated with a mixed ANOVA with between-subjects factor of genotype and within-subjects factor of region. Change from 0 was calculated with a two-sided one-sample *t* test (SciPy.stats, ttest_1samp), which was corrected with the Benjamini–Hochberg (BH) method.

### Functional connectivity and graph theory analysis

For functional analysis, a high-level parcellation scheme was applied to segment 36 unilateral regions (18 per hemisphere) excluding white matter from the acquired 3D volume. The BOLD signal time-courses were averaged within each ROI, and the mean time-courses were extracted using FSL’s fslmeants tool. Pearson correlation coefficients were calculated for each time-course pair, producing a 36×36 correlation matrix for each subject. These matrices were analysed as functional connectivity (FC) graphs, with edge strength determined by the Fisher z transformed Pearson correlation coefficient. To identify the strongest connections, graphs were thresholded at 5% intervals from 5% to 50%. At each threshold level, referred to as the graph sparsity interval, connections below threshold were set to zero, generating 10 sparsity graphs per subject. FC was calculated as the average non-zero connectivity at each threshold level. Global graph metrics, global efficiency and clustering coefficient were computed at each sparsity level using Brain Connectivity Toolbox algorithms^55^ implemented with Network X (3.4.2) global_efficiency and clustering, passing thresholded Pearson matrices. The area under the curve (AUC) was computed per subject using trapezoidal numerical integration (numpy.trapz), and the likelihood of observed values was estimated with permutation testing (10 000 permutations).

To estimate changes over time, global graph metric values at adolescence were subtracted from adulthood values at each sparsity interval. Group differences were again tested by calculating the AUC and applying permutation testing. Changes from baseline (zero) were evaluated using two-sided one-sample *t* tests (difference from 0), corrected for multiple comparisons with the BH method.

### FDR correction and network-based statistics

Matrices for *C3ar1*-deficient and wild-type mice were compared with Student’s *t* tests for each pairwise connection, resulting in 36×36 *t*-statistic and *P*-value matrices. To control for multiple comparisons, FDR correction was applied to the upper triangle of the *P*-value matrix using the BH procedure.

For network-based statistics (NBS), the above-described *t* matrices were thresholded at |*t*| ≥ 2. To identify connected components within the FC graph, adjacency matrices representing significant connections (|*t*| ≥ 2) were converted into graph objects using NetworkX. Regions of interest (ROIs) were treated as nodes, and significant connections as edges. Connected components were identified using a breadth-first search (BFS) algorithm, which explores all neighbouring nodes before moving deeper into the graph. Only components containing more than one ROI were retained for further analysis. To generate a null distribution of maximal component sizes, group labels were randomly shuffled across subjects for each permutation while preserving matrix structure (10 000 permutation). The *P*-value for an observed component was calculated as the proportion of permutations where the maximal component size exceeded that of the observed component.

### *A priori* node strength analysis

We selected 20 (10 left + 10 right) anxiety and fear related regions (cingulate cortex, prefrontal cortex, amygdala, pallidum and nucleus accumbens, striatum, hypothalamus, dorsal hippocampus, ventral hippocampus, periaqueductal grey, brain stem) and calculated their average absolute connectivity to all other regions using Fisher z-transformed Pearson correlation coefficients. We then used a mixed ANOVA with between-subjects factor of genotype and within-subjects factor of region followed by pairwise testing with the BH method.

### Within-network analysis

Two mouse resting state networks were subset from correlation matrices: the default mode network (DMN) and the salience network.^56,57^ The DMN included bilateral prefrontal cortex, cingulate cortex, and dorsal hippocampus, while the salience network comprised bilateral cingulate cortex, amygdala, and striatum. Additionally, a third anxiety-related network was defined, consisting of the 20 regions described in the node-strength analysis above.

For each network, mean FC was calculated as the average of all pairwise connections between nodes within the network, without applying a threshold. For individual network global efficiency analysis, thresholding was not used due to the small number of nodes. Instead, Pearson matrices normalized to range from 0–1 were passed to bctpy (0.6.1) efficiency_wei computing weighted efficiency.

Student’s *t* tests were used to test significance, and the comparisons were corrected within outcome measure with the BH method.

### Voxel-wise seed-to-brain analysis

We conducted voxel-wise seed-based FC analyses for anxiety-related regions, as well as the colliculus and sensory cortex, which served as control regions not specific to anxiety. For each seed region, the time-course of the BOLD signal was extracted and regressed with the BOLD signal of every voxel in the brain, resulting in a 3D spatial map of the connectivity with the seed. Group-level comparisons of these maps were performed between genotypes using voxel-wise permutation tests with FSL’s randomize (5000 permutation), converted with TFCE and corrected for multiple comparisons using FWE (seed-to-brain), but these were not further corrected for the presence of multiple seeds.

### Statistical procedure for behavioural outcome measures

All statistical analyses were conducted using Python 3.11.7, using relevant libraries such as SciPy and statsmodels. For both studies, the Shapiro-Wilk test was used to assess the normality of data distributions, while Levene’s test was used to evaluate homogeneity of variances. When either assumption of normality or equal variance was violated, the Kruskal-Wallis test was used, followed by Dunn’s test for post hoc pairwise comparisons with Bonferroni correction for multiple testing where applicable. For datasets meeting parametric assumptions, different approaches were used based on the study design. For Cohort 1, a two-way ANOVA was performed to analyse the effect of sex, genotype and sex-by-genotype interaction, followed by Tukey’s test for post hoc comparisons. Bonferroni correction was applied to adjust for multiple comparisons. Due to the relatively small sample size (*N* = 38) in Cohort 2, sex-by-genotype interactions were not analysed. Instead, Student’s *t* tests were used for comparisons between genotypes.

To balance statistical rigour with preserving power in exploratory contexts, behavioural measures were not universally corrected for multiplicity. Instead, robustness was inferred through replication across independent cohorts and broad phenotypic consistency. The threshold for statistical significance was set at *P* < 0.05. Individual mice served as the experimental units in all analyses.

## Results

### *C3ar1^tm1Cge/tm1Cge^* mice lack detectable *C3ar1* mRNA

**Figure 1:**
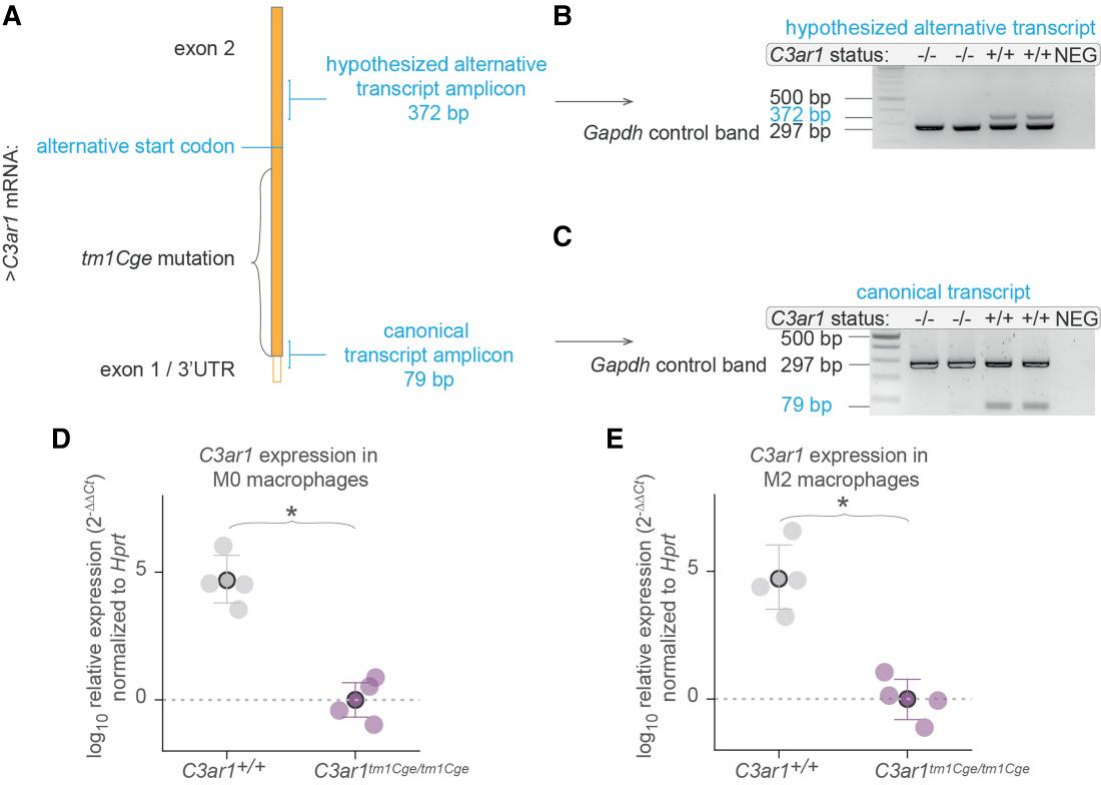
***C3ar1^tm1Cge/tm1Cge^* mice lack detectable *C3ar1* mRNA.** (**A**) Schematic shows *C3ar1* mRNA with its only protein coding exon and the design of the 79 bp PCR amplicon which targets an exon-1/5’ untranslated region (UTR) and exon-2 junction of the canonical *C3ar1* wildtype transcript. Also shown is another 372 bp amplicon which targets a region downstream of the deletion and after an alternative start codon. The start of exon 2 is deleted in *C3ar1^tm1Cge/tm1Cge^* mice, so PCR should result in no amplification. Similarly, if no alternative transcript is present, there should be no amplification. (**B–C**) Gel images show PCR products of cDNA from bone-marrow derived interleukin 4 (IL4)-induced M2-like macrophages. Each sample well has a 297 bp Glyceraldehyde 3-phosphate dehydrogenase (*Gapdh*) control band. -/- = *C3ar1^tm1Cge/tm1Cge^*, +/+ = *C3ar1^+/+^*. NEG: no reverse transcriptase negative control carried over from cDNA synthesis. (**B**) Canonical transcript. (**C**) Hypothesized alternative transcript. The bands appear hollow due to over-saturation. Original uncropped gel images can be found in Supplemental figure 3. (**D**, **E**) qPCR of cDNA from bone marrow-derived macrophages: (**D**) M0 and (**E**) M2 from *C3ar1^tm1Cge/tm1Cge^* mice and wildtype littermates (*N* = 4 mice for each genotype), Kruskal Wallis test M0 *P* < 0.05, M2 *P* < 0.05. Point plots show mean ± 95% CI, data points for individual mice are shown. *Hprt* = Hypoxanthine-Guanine Phosphoribosyltransferase 1 (housekeeping gene).

We used an established *C3ar1* mutant line, *C3ar1^tm1Cge^,*^45^ and performed our own validation of the mutation. For this purpose, we designed a 79-base pair (bp) amplicon targeting the putatively deleted region (Fig. 1A). PCR analysis of cDNA derived from bone marrow-derived macrophages—a cell type consistently reported to express high levels of *C3ar1* mRNA^58-60^—showed no detectable transcript in this region in the mutant animals (Fig. 1C), confirming the absence of the canonical transcript. The *C3ar1* gene contains an in-frame start codon after the deletion (Supplemental Fig. 2**)**. We also confirmed that no transcript is made that includes this region (Fig. 1B). These results were further corroborated by quantitative PCR (qPCR, *N =* 8) in homozygous knockout animals in either M0-like (Fig. 1D) or interleukin 4 (IL4)-induced M2-like macrophages (Fig. 1E, see Supplemental Figs 3C–E for macrophage polarization confirmation). Together, these results confirm that *C3ar1^tm1Cge^* is a true loss-of-function or a ‘knockout’ allele resulting in no detectable *C3ar1* transcript.

### *C3ar1*-deficiency does not influence total or regional brain volume

**Figure 2:**
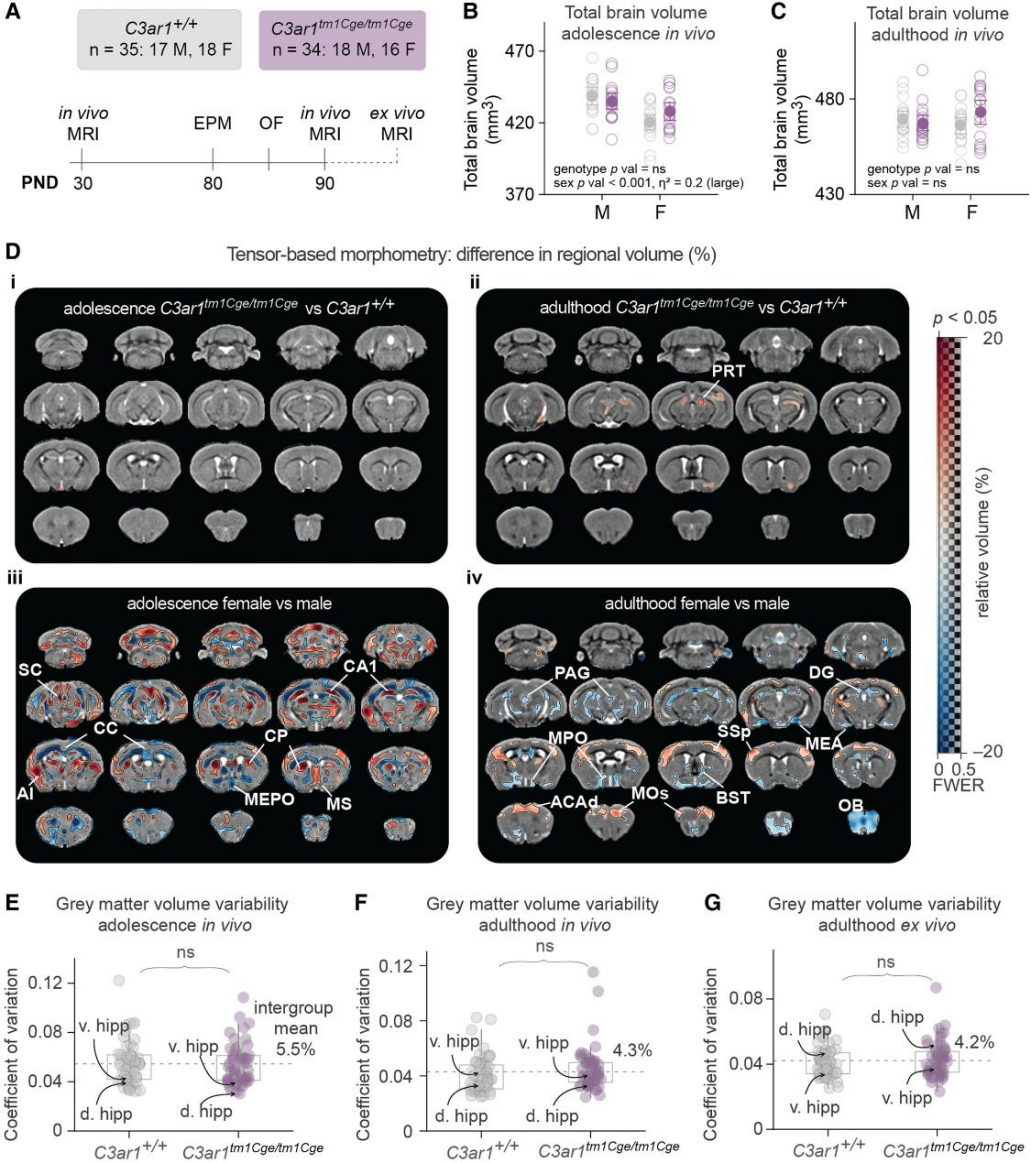
**Sex but not *C3ar1* status influences regional brain volume.** (**A**) Schematic of the MRI study shown in (**D–G**). 69 mice were scanned twice *in vivo*; in adolescence at ∼postnatal day (PND) 30 (range 27–31) and at adulthood ∼PND90 (range 81–92), as well as once *ex vivo* after sacrifice following the adulthood scanning session. Behavioural tests were carried out before the adulthood scanning session. EPM = elevated plus maze, OF = open field. (**B**) Total brain volume in adolescence. Two-way ANOVA, genotype, sex, genotype * sex, F_[1,65]_ = 0.35, 16.72, 3.26, *P* = 0.56, <0.001, 0.76. (**C**) Total brain volume in adulthood. Two-way ANOVA, genotype, sex, genotype * sex, F_[1,65]_ = 0.74, 0.26, 3.33, *P* = 0.39, 0.61, 0.07. (**B–C**) Data presented as mean ± 95% CI. Each datapoint represents an individual mouse. (**D**) Panels showing relative regional volume changes (%) overlaid on study-specific coronal templates (grey). Red hues signify areas larger in *C3ar1^tm1Cge/tm1Cge^* (**i–ii**) or females (**iii–iv**) and blue hues signify areas larger in *C3ar1^+/+^* (**i–ii**) or males (**iii–iv**). Transparency of the colour overlay shows the statistical significance, ranging from family-wise error rate (FWER)-corrected voxel-wise *t* test *P* value 0.5 to 0 (transparent to opaque, respectively). Areas where FWE-corrected *P* value < 0.05 are demarcated with a black line, and where the *P* value > 0.5 are grey (no overlay), meaning that in adolescence genotype comparison (**i**), no voxels had a *P* value < 0.5. The locations of the coronal slices in relation to bregma in the left most column from top: −7.6, −4.6, −1.6, 1.4 mm. *C3ar1^+/+^ N* = 35, 17 males and 18 females; *C3ar1^tm1Cge/tm1Cge^ N* = 34, 18 males and 16 females. ACAd = dorsal anterior cingulate cortex, AI = agranular insular cortex, BST = bed nucleus of stria terminalis, CA = cornu ammonis, CP = caudoputamen, DG = dentate gyrus, MEA = medial amygdala, MEPO = medial preoptic nucleus, MOs = secondary motor cortex (MOs), MPO = medial preoptic area, MS = medial septum, OB = olfactory bulb, PAG = periaqueductal grey, PRT = pretectal area, SC = superior colliculus, SSp = primary somatosensory cortex. (**E**) Regional volume coefficient of variation (CV) in adolescence *in vivo*. (**F**) CV in adulthood *in vivo*. (**G**) CV in adulthood *ex vivo*. (**E–G**) Each data point represents the CV calculated for an individual brain region, using data from all animals; thus, *N* = 66 regions (all atlas regions excluding cerebrospinal fluid areas). Data are shown with quartiles, whiskers show the extent of the distribution. Kruskal Wallis *P* values, all ns. D. hipp = dorsal hippocampus, v. hipp = ventral hippocampus.

We conducted a longitudinal MRI study (referred to as Cohort 1 hereafter or implied when cohort is not specified) to investigate potential genotype-related differences in brain structure and function using *C3ar1^tm1Cge/tm1Cge^* homozygous knockout mice (also referred to as *C3ar1*-deficient) and their littermate wild-type controls (*C3ar1^+/+^*) (Fig. 2A) on C57BL6J (Charles River, UK) background. Both groups underwent *in vivo* MRI in adolescence (PND27–31) and adulthood (PND81–92). Structural MR images were additionally collected *ex vivo* from the same mice sacrificed in adulthood immediately after the *in vivo* scan to achieve higher isotropic resolution (0.1 mm *ex vivo* versus 0.15 mm *in vivo*) and increased signal-to-noise ratio (SNR). To corroborate these results, we also conducted MRI in adulthood (PND74-110) in an independent study cohort, referred to as Cohort 2.

We used tensor-based morphometry (TBM) analysis to estimate total brain volume and to map regional brain volume differences. There were no genotype-dependent differences in total brain volume *in vivo* in adolescence (Fig. 2B) nor in adulthood (Fig. 2C). In all adolescent mice, irrespective of genotype, female mice exhibited significantly smaller total brain volumes compared to males. These sex differences disappeared by adulthood—a finding that aligns with previous observations in wild-type mice.^61^

Still using TBM, no significant genotype-dependent differences in regional brain volumes were detected in adolescence (Fig. 2Di). In adulthood (Fig. 2Dii), *C3ar1^tm1Cge/tm1Cge^* mice showed a significant (TFCE-corrected *P* < 0.05) volume increase in the right pretectal area (72 voxels), and subthreshold (0.05 < TFCE-corrected *P* < 0.5) increases in the left pretectal area (111 voxels) and the right lateral thalamus *in vivo*, but these differences were no longer observed in the same study cohort *ex vivo* despite improved spatial resolution (not shown since these group-level data would be presented as empty study-template maps), nor did we observe any genotype-dependent differences using TBM analysis in Cohort 2 (not shown). We also did not observe any significant genotype-by-sex interaction effects (not shown).

While no reproducibly significant genotype effects in regional volume were observed in Cohort 1, we nevertheless detected sexually dimorphic effects (Fig. 2**Diii** and 2**d-iv**). Adolescent female mice had significantly larger volumes (relative to total brain volume) bilaterally in the agranular insular cortex (AI), superior colliculus (SC), medial septum (MS) and in the CA1 region of the hippocampus, whereas male mice had increased relative volumes in white matter areas including the olfactory tract, corpus callosum, hippocampal commissure, and notably also in the median preoptic nucleus (MEPO) which is well known to be larger in male rodents^62^ (Fig. 2**Diii,**  Supplemental Fig. 4 for absolute volume). Echoing total brain volume measures, many of these sex differences were no longer observed in adulthood (see also Supplemental Fig. 5 for Cohort 2 data), but females showed larger relative volumes in the dorsal anterior cingulate cortex (ACAd), secondary motor cortex (MOs) and primary somatosensory cortex (SSp). Adult males had larger relative volumes in areas including the medial amygdala (MEA) and the bed nucleus of stria terminalis (BST) which, like the MEPO, are previously documented sexual dimorphisms^63^ and which were also observed *ex vivo* in this study cohort (Supplemental Fig. 6).

There were no genotype nor sex-by-genotype interactions on regional brain volumes at either time-point (not shown). Overall, female somatosensory and motor cortices increased more in volume between adolescence and adulthood than male (Supplemental Fig. 7), in line with the observed smaller differences in these areas in adulthood compared to adolescence.

If variability was high within our study sample, particularly in the *C3ar1*-deficient group, it would have hampered our ability to detect significant differences. The coefficient of variation for grey matter region-of-interest (ROI) volume showed no differences between genotypes *in vivo* in adolescence (Fig. 2E) or adulthood (Fig. 2F), nor *ex vivo* in adulthood (Fig. 2G). The overall variability was low, ranging from 4.2–5.5% *in vivo* and 4.2% *ex vivo*, while hippocampal variability which has previously been repeatedly measured establishing a neuroimaging gold standard at 5% variability,^53^ was 3.7–3.8% *in vivo* and 4.2% *ex vivo* in our study. These values suggest that our study was well-positioned to detect a genotype effect if one had been present.

### *C3ar1*-deficiency does not influence fractional anisotropy

**Figure 3:**
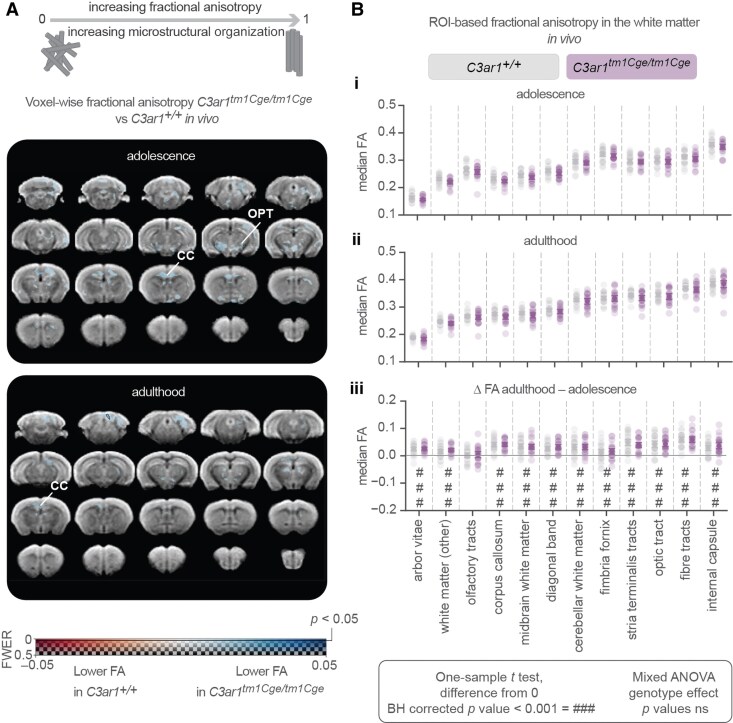
**Fractional anisotropy does not depend on *C3ar1* status but increases with age.** (**A**) Panels showing voxel-wise fractional anisotropy analysis (voxel-wise *t* tests) in adolescence (top) and adulthood (bottom) corrected for FWER. There are no significant voxels (no black contour) where genotype effect is significant (*P* < 0.05). CC = corpus callosum, OPT = optic tract. (**B**) *In vivo* ROI-based median fractional anisotropy values in white matter regions for (**i**) adolescence, (**ii**) adulthood and **(iii)** change over time (adulthood − adolescence). Two-sided one-sample *t* test (difference from 0) *P* values that were adjusted with Benjamini-Hochberg (BH) procedure (### *P* value < 0.001). (**Bi–iii**) Mixed ANOVA with genotype and genotype-by-region interaction effects, all ns. Data are presented as mean ± 95% CI. (**A–B**) Adolescence: *C3ar1^+/+^ N* = 34, 16 males and 18 females; *C3ar1^tm1Cge/tm1Cge^ N* = 33, 17 males and 16 females. Adulthood: *C3ar1^+/+^ N* = 31 (15 males and 16 females); *C3ar1^tm1Cge/tm1Cge^ N* = 26 (13 males and 13 females). Change: *C3ar1^+/+^ N* = 30 (14 males and 16 females); *C3ar1^tm1Cge/tm1Cge^ N* = 25 (12 males and 13 females).

To assess the potential impact of *C3ar1* deficiency on white matter integrity, we measured fractional anisotropy as an indirect marker of axonal microstructure^64^ (Fig. 3). Using voxel-wise analysis, we observed sub-threshold, non-significant (0.05 < *P* < 0.5) decreases in fractional anisotropy in *C3ar1*-deficient mice compared to wild-types (Fig. 3A). In adolescence, sub-threshold reductions were noted in the corpus callosum (CC) and optic tract (OPT, Fig. 3A, upper panel). In adulthood, they were primarily localized to the CC (Fig. 3A, bottom panel). These sub-threshold differences were not observed *ex vivo*. Compared to males, female mice had sub-threshold decreases in fractional anisotropy *in vivo* in adulthood in the internal capsule and in the third ventricle (Supplemental Fig. 8), but this was again not observed *ex vivo* (not shown). We did not observe any sex-by-genotype interactions in these datasets (not shown).

In our ROI-based fractional anisotropy analysis, we focused on predefined white matter regions, hypothesizing that fractuonal anisotropy alterations would primarily occur in areas containing axonal tracts due to increased microglial phagocytosis in *C3ar1*-deficient mice during development.^21,40,41^ In this analysis also, no significant genotype effects were observed in adolescence (Fig. 3B**i**) or adulthood (Fig. 3B**ii**). There were no genotype-dependent differences in the change in fractional anisotropy over time, but it increased between adolescence and adulthood in all white matter areas except the olfactory tract (Fig. 3B**iii**), which is in line with the reported early maturation of the olfaction system in mice.^65^

### *C3ar1*-deficiency does not influence global functional brain connectivity

**Figure 4:**
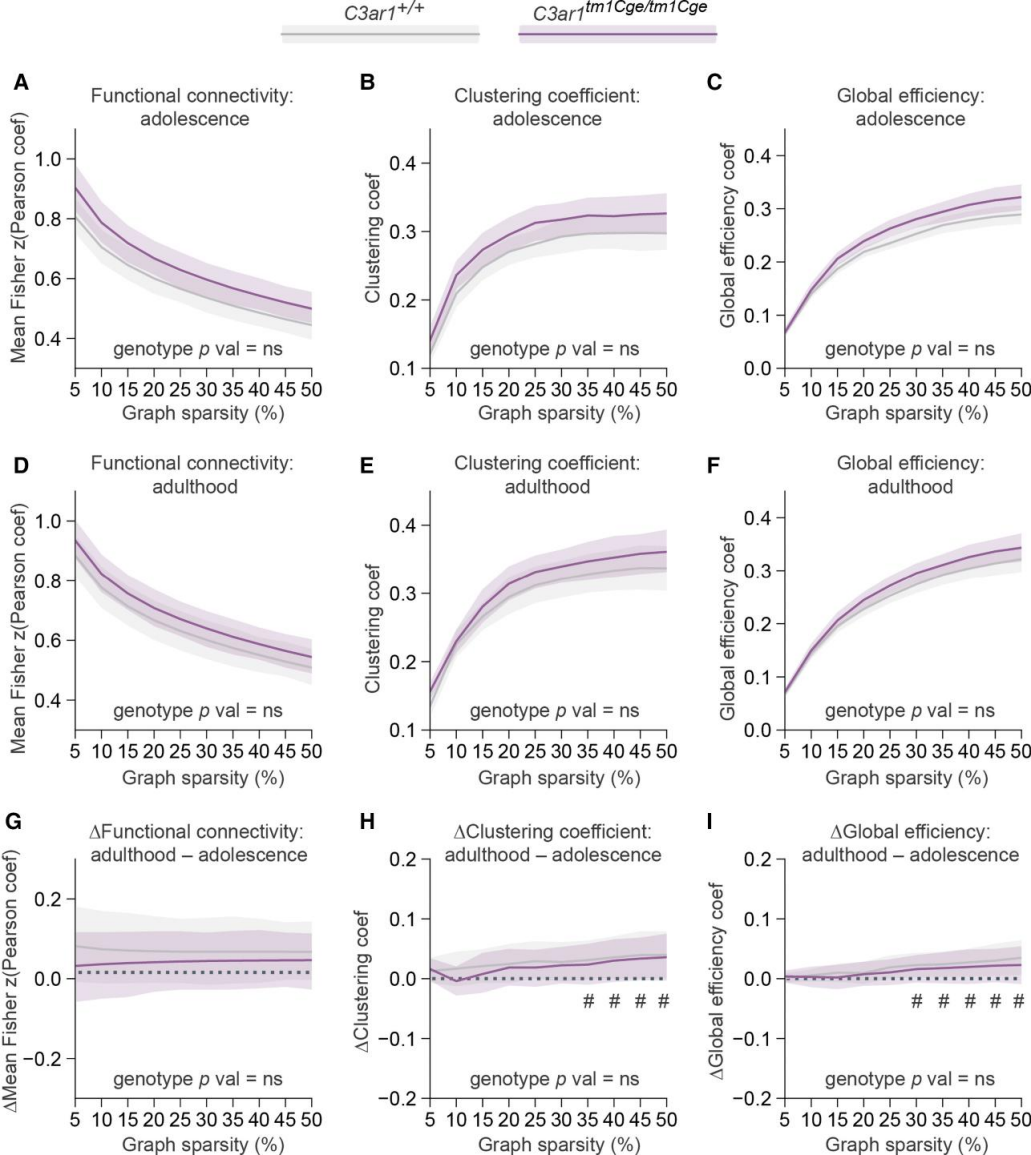
**Global functional connectivity is not changed in *C3ar1*-deficient mice.** Functional connectivity, clustering coefficient and global efficiency at decreasing graph sparsity levels in adolescence (**A–C**), and adulthood (**D–F**), and change over time (**G–I**). (**A–I**) Statistical significance was determined with sexes combined using AUC permutation testing (10 000 iterations) and the resulting *P* values were corrected for multiplicity with the Bonferroni method (*N* = 3 tests per outcome measure). Adolescence: *C3ar1^+/+^ N* = 32 (15 males, 17 females); *C3ar1^tm1Cge/tm1Cge^ N* = 32 (18 males, 14 females); Adulthood: *C3ar1^+/+^ N* = 33 (16 males, 17 females) and *C3ar1^tm1Cge/tm1Cge^ N* = 32 (17 males, 15 females); change: *C3ar1^+/+^ N* = 30 (14 males and 16 females); *C3ar1^tm1Cge/tm1Cge^ N* = 31 (17 males and 14 females). Data are shown as mean ± 95% CI. (**G–I**) Two-sided one-sample *t* tests (difference from 0) for global connectivity changes at each sparsity level with genotypes combined, corrected using the BH procedure (*N* = 10 sparsity levels), # = adjusted *P* value < 0.05. Coef = coefficient.

Functional connectivity (FC) changes have been reported for other microglial receptor knockouts, including triggering receptor expressed on myeloid cells 2 (*Trem2)*, CX3C motif chemokine receptor 1 (*Cx3cr1)*, and complement receptor 3 (*Cr3)*.^66-68^ Specifically, *Trem2* and *Cx3cr1* knockout mice exhibited impaired synapse elimination in these studies, alongside decreased FC, social behaviour deficits and increased repetitive behaviour.^66,68^ In contrast, *Cr3* knockout mice did not show deficits in synapse or axon refinement but showed reduced phagocytosis of perinatal cortical neurons and higher cortical FC.^67^ Since *C3ar1*-deficiency appears to cause a deficit of developmental astrocyte phagocytosis, at least in the retina,^21^ it is plausible that a similar mechanism could lead to increased cellular phagocytosis also in the brain parenchyma, as seen with *Cr3* knockouts. This may result in a higher number of neurons during early embryonic development, contributing to increased FC. We estimated FC through analysis of BOLD signal time-courses with the assumption that the magnitude of correlation between these time-courses corresponds to the strength of FC. We calculated pairwise correlation coefficients between 36 (18 per hemisphere) grey matter regions. Non-zero correlation values were averaged across proportional progressively decreasing sparsity thresholds to preserve biologically meaningful weak correlations while minimizing noise.^69^ No genotype-dependent differences in global FC were observed in adolescence (Fig. 4A), adulthood (Fig. 4D) or in the change over time (Fig. 4G).

We applied graph theory to characterize global brain network connectivity across all regions, focusing on two key metrics: clustering coefficient (Fig. 4B and E and H) and global efficiency (Fig. 4C, F and I), and applying the same strategy for proportional thresholding as for FC. The clustering coefficient reflects the tendency of nodes to form connected local clusters, with higher values indicating the presence of more highly interconnected subnetworks within the brain.^70^ Global efficiency refers to the average of shortest paths linking nodes in a network and can be a proxy of information integration abilities since it decreases with cognitive deficit^71,72^ and increases with development.^73^ Clustering coefficient and global efficiency appeared higher in *C3ar1*-deficient animals at both time-points, but this was not significant (Supplemental table 1**)**. Further, we found no effects of genotype across global connectivity measures when we treated males and females as separate groups (Table 1, for *P* values, see Supplemental table 2).

**Table 1.** Mean network connectivity metrics ± 95% CI in males and females of both genotypes

Age	Adolescence				Adulthood			
*C3ar1* status	+/+	-/-	+/+	-/-	+/+	-/-	+/+	-/-
**Sex**	F	F	M	M	F	F	M	M
**Connectivity metric (AUC** ± **95% CI)**								
FC	25.5 ± 2.0	27.5 ± 3.1	25.9 ± 5.0	29.6 ± 4.5	27.4 ± 3.5	29.3 ± 3.0	30.2 ± 5.7	31.9 ± 4.7
GE	10.3 ± 0.6	11.2 ± 0.8	10.1 ± 0.9	11.3 ± 1.1	10.6 ± 0.8	11.4 ± 0.9	11.4 ± 1.2	12.2 ± 1.3
CC	12.0 ± 0.9	13.0 ± 1.3	12.0 ± 1.9	13.4 ± 1.8	12.8 ± 1.4	13.8 ± 1.4	13.6 ± 2.0	14.4 ± 1.8

FC = functional connectivity, GE = global efficiency, CC = clustering coefficient

Global efficiency and clustering coefficient appeared to increase with brain maturation when genotype groups were combined, but this was only significant when graph sparsity was lower, that is, when 30–50% of the strongest connections were retained, with small effect sizes observed (Cohen’s *d* = 0.30–0.32). These findings indicate that while *C3ar1* deficiency did not result in alterations in global network properties under our experimental conditions, developmental changes in global efficiency and clustering coefficient were detectable.

### *C3ar1*-deficiency does not influence functional brain networks

**Figure 5:**
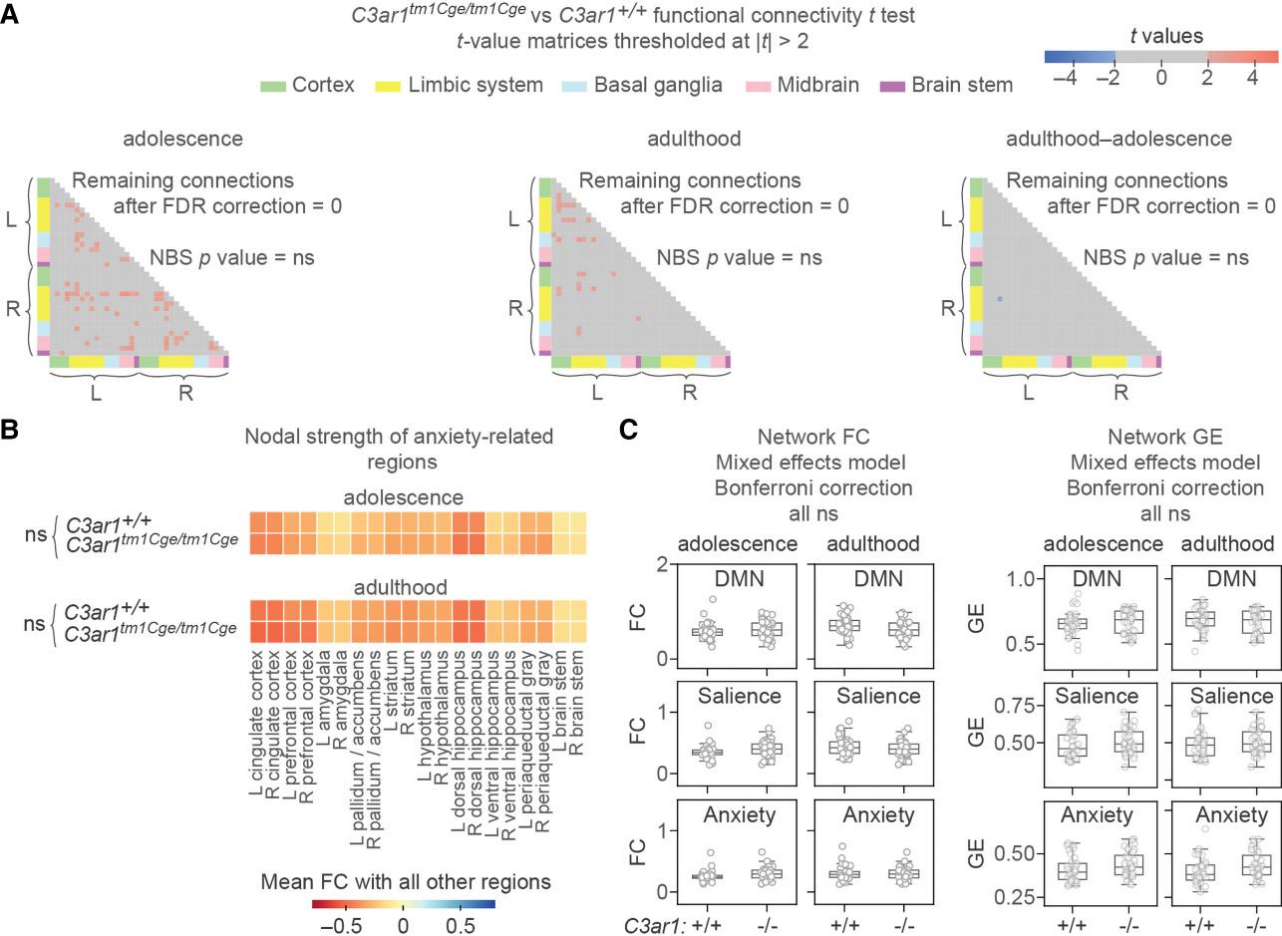
***C3ar1*-deficiency does not influence functional brain networks.** (**A**) Thresholded adjacency matrices comparing *C3ar1^+/+^* and *C3ar1^tm1Cge/tm1Cge^* mice where |*t*| ≥ 2 in adolescence and adulthood, and the change between time points. No connections with |*t*| ≥ 2 remained significant after the false discovery rate (FDR) correction. Additionally, the number of connections in the |*t*| ≥ 2 adjacency matrix components were not significant when assessed using network-based statistics (NBS) correction. (**B**) Nodal strength (mean absolute connectivity) of *a priori* anxiety-related seeds (e.g. L cingulate cortex correlation coefficients with all other regions). *P* values were calculated using a mixed ANOVA with between-subjects factor of genotype and within-subjects factor of region. Only the region effect was significant at both time-points (both *P* values < 0.001, η_p_^2^ = 0.69 in adolescence and η_p_^2^ = 0.73 in adulthood). (**C**) Functional connectivity (FC) and global efficiency (GE) were analysed using mixed effects linear models with genotype, time point (adolescence and adulthood) and their interaction as fixed effects and mouse ID as a random effect. Analyses were run separately for each network, corrected with the Bonferroni method (three tests). All *P* values = ns. (**A–C**) Adolescence: *C3ar1^+/+^ N* = 32 (15 males, 17 females); *C3ar1^tm1Cge/tm1Cge^* N = 32 (18 males, 14 females); Adulthood: *C3ar1^+/+^ N* = 33 (16 males, 17 females) and *C3ar1^tm1Cge/tm1Cge^ N* = 32 (17 males, 15 females).

Since *C3ar1*-deficient mice did not show statistically significant changes in global connectivity metrics, we next examined specific networks after conducting *t* tests for each pairwise ROI FC between genotypes. For this we used two hypothesis-free approaches; false discovery rate (FDR) correction to identify strongly differing edges between genotypes, and network-based statistics (NBS)^74^ to detect network-level differences while controlling for family-wise error rate. We thresholded the resulting *t*-value matrices at |*t*| ≥ 2 for adulthood and adolescence, and their changes over time (adulthood—adolescence; Fig. 5A). However, no edges remained significant after FDR correction. Then, using NBS which tests the likelihood of detecting a connected component of a specific size, we determined that the network sizes observed after thresholding at |*t*| ≥ 2 (*N* = 68 at adolescence, *N* = 15 at adulthood, and *N* = 1 for change) could occur by chance in these datasets.

Given that neither one of the hypothesis-free approaches, FDR correction and NBS, detected any genotype-related differences, we next focused on anxiety-related regions. This decision was motivated by prior evidence of anxiety-like behaviour in *C3ar1*-deficient animals^30^ and the inclusion of anxiety-specific tests in our behavioural battery. We calculated the mean FC, or nodal strength, of 20 *a priori* selected brain regions relevant to anxiety. We did not detect effects of genotype (Fig. 5B), sex (**Table 2**) or genotype-by-region interaction (Supplemental table 3).

**Table 2.** Mean anxiety-associated node connectivity in males and females of both genotypes

Age	Adolescence				Adulthood			
*C3ar1*status	+/+	-/-	+/+	-/-	+/+	-/-	+/+	-/-
Sex	F	F	M	M	F	F	M	M
**Brain area**	**Mean node connectivity strength**							
L cingulate cx	0.39	0.41	0.39	0.44	0.45	0.49	0.48	0.54
R cingulate cx	0.38	0.41	0.38	0.43	0.45	0.49	0.48	0.53
L prefrontal cx	0.29	0.33	0.32	0.37	0.41	0.44	0.37	0.49
R prefrontal cx	0.28	0.28	0.33	0.37	0.41	0.45	0.39	0.47
L amygdala	0.14	0.17	0.15	0.2	0.2	0.2	0.24	0.28
R amygdala	0.11	0.16	0.15	0.18	0.19	0.2	0.25	0.25
L pallidum & accumbens	0.23	0.27	0.23	0.31	0.3	0.29	0.35	0.4
R pallidum & accumbens	0.24	0.26	0.24	0.3	0.31	0.31	0.35	0.39
L striatum	0.28	0.32	0.3	0.36	0.32	0.36	0.4	0.43
R striatum	0.26	0.31	0.32	0.35	0.33	0.36	0.42	0.41
L hypothalamus	0.28	0.29	0.27	0.34	0.32	0.32	0.34	0.38
R hypothalamus	0.25	0.27	0.26	0.32	0.31	0.32	0.33	0.37
L dorsal hippocampus	0.4	0.43	0.42	0.49	0.44	0.46	0.48	0.52
R dorsal hippocampus	0.41	0.43	0.42	0.49	0.44	0.47	0.5	0.52
L ventral hippocampus	0.18	0.18	0.17	0.26	0.21	0.23	0.2	0.27
R ventral hippocampus	0.18	0.23	0.19	0.25	0.22	0.25	0.25	0.26
L PAG	0.28	0.3	0.3	0.38	0.27	0.3	0.32	0.36
R PAG	0.28	0.31	0.3	0.39	0.27	0.29	0.32	0.37
L brain stem	0.11	0.11	0.12	0.18	0.1	0.14	0.16	0.16
R brain stem	0.11	0.09	0.12	0.18	0.11	0.13	0.17	0.18

Next, we examined FC and global efficiency within two resting state networks that have been linked to anxiety and emotionality in humans^75-77^ and that have been observed in mice^56,57,78^; the default mode network (DMN) and the salience network (Fig. 5C). Additionally, we analysed network connectivity within a third, anxiety network (regions in Fig. 5B). Consistent with our earlier findings, we did not observe genotype-dependent differences in these networks at either time-point.

**Figure 6:**
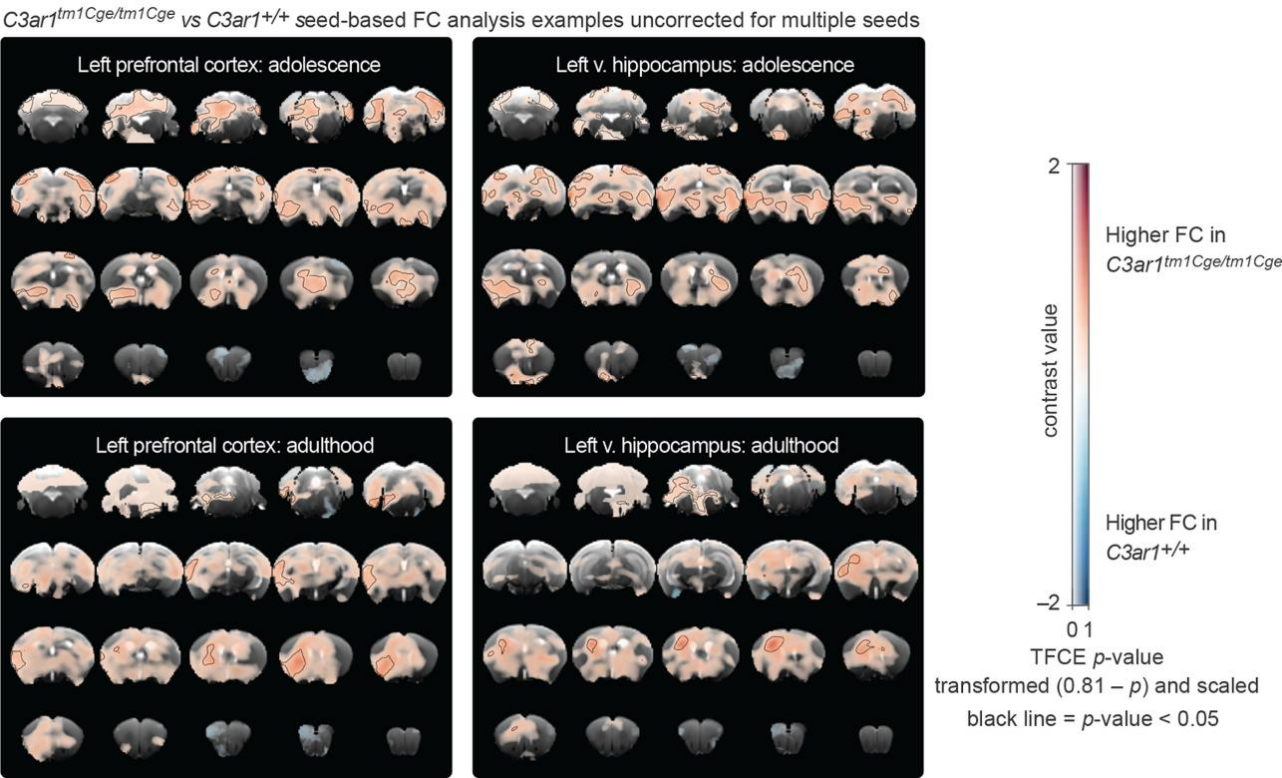
**Examples of seed-based FC maps.** Shown are voxel-wise group differences between *C3ar1^tm1Cge/tm1Cge^* and *C3ar1^+/+^* mice (using *t* tests) with seeds placed in the left ventral hippocampus and left prefrontal cortex in adolescence and adulthood datasets. The dual scale bar displays contrast value on the x-axis and TFCE *P*-values (transformed 0.81—*P*) on the y-axis. The transformed *P*-values have been re-scaled to range from 0 to 1 for visualisation, with darker colours representing greater statistical significance. Black outlines demarcate regions where TFCE *P*-values are below 0.05. Adolescence: *C3ar1^+/+^ N* = 32 (15 males, 17 females); *C3ar1^tm1Cge/tm1Cge^* N = 32 (18 males, 14 females); Adulthood: *C3ar1^+/+^ N* = 33 (16 males, 17 females) and *C3ar1^tm1Cge/tm1Cge^ N* = 32 (17 males, 15 females).

Significant genotype-related differences were detected only in our voxel-wise seed-to-brain connectivity analysis (Fig. 6). However, these findings should be interpreted with caution, as the analysis controlled for voxel-wise comparisons within subjects but did not correct for multiple seed tests, which limits the robustness of the results. Nevertheless, some anxiety-related regions—for example, the left prefrontal cortex and left ventral hippocampus (see Supplemental Fig. 9 for the right hemisphere)—showed weak and widespread increases in connectivity in *C3ar1*-deficient animals compared to controls at both time-points. Seed-based analyses also revealed higher functional connectivity across the brain when other anxiety-related regions were used as seeds, being most pronounced during adolescence (Supplemental Fig. 10). However, this effect was not confined to anxiety-related regions or specific networks (Supplemental Fig. 11). Therefore, being non-specific and weak, these findings cannot at this point be clearly distinguished from noise.

### *C3ar1*-deficient mice do not have discernible behavioural phenotypes

Behavioural testing is another way of assessing functional consequences of genetic manipulations with expected neurodevelopmental sequelae.^79^ We aimed to evaluate the impact of *C3ar1* deficiency on anxiety-like behaviour, locomotion and recognition memory, which were chosen based on prior reports of abnormalities in *C3ar1*-deficient mice. We used a battery of well-established behavioural tests, including the OF test and EPM for anxiety-like behaviour and locomotion, as well as NOR for recognition memory. We also tested PPI which is a sensorimotor reflex consistently found to be attenuated in schizophrenia^80,81^ but which has hitherto not been tested in *C3ar1*-deficient mice.

We performed behavioural testing in both cohorts described in the structural MRI results sections. In Cohort 1 (Fig. 2A), behavioural testing (EPM, then OF) occurred shortly before the adulthood MRI scan (and two months after adolescence MRI). Behaviour of Cohort 2 was also tested in adulthood (OF, NOR, EPM, PPI, in that order) followed by MRI, except that these mice were not previously scanned under anaesthesia in adolescence. Behavioural data from each cohort were analysed separately to account for differences in study design.

**Figure 7:**
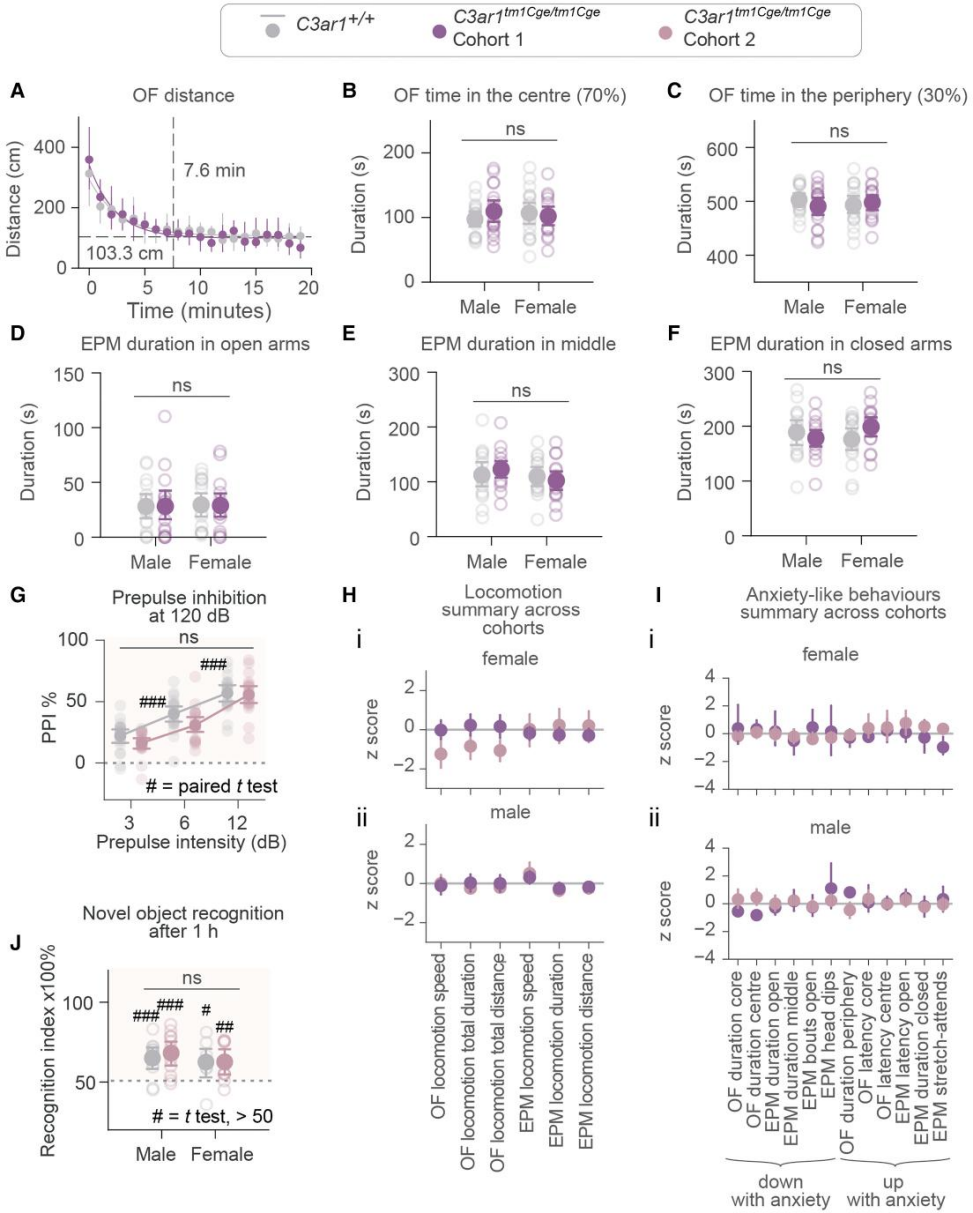
***C3ar1* deficiency does not cause behavioural abnormalities.** (**A**) Cohort 1 OF distance travelled in one-minute bins plotted as median ± interquartile range, fitted with exponential decay curves; dotted lines show the asymptote and the time animals reach 95% stability in distance travelled per minute. Effects of time bin, genotype and their interaction tested with two-way ANOVA; only time was significant (*P* < 0.01). (**B–F**) Cohort 1 OF and EPM: anxiety-like outcomes for *C3ar1^+/+^* and *C3ar1^tm1Cge/tm1Cge^* mice, separated by sex. Statistical comparisons made using two-way ANOVA (genotype × sex) for each metric; all effects ns so no post-hoc comparisons were performed. Individual *P* values can be found in Supplemental table 5. (**G**) Prepulse inhibition (PPI) in Cohort 2. Individuals plotted per genotype, and sexes are separated only for visualization. PPI increased with increasing prepulse intensity in both genotypes (paired *t* tests *P* value ### < 0.001); no genotype differences by Student’s *t* tests (*C3ar1^+/+^* versus *C3ar1^tm1Cge/tm1Cge^* all *P* values > 0.05). Dashed line marks inhibition threshold. (**H–I**) Z-scored locomotion and anxiety metrics for *C3ar1^tm1Cge/tm1Cge^* mice normalized to *C3ar1^+/+^* mean, showing Cohorts 1 and 2. (**J**) Novel object recognition recall 1 h after acquisition in Cohort 2. Sexes are separated only for visualization. Both genotypes showed novelty preference (one-sample *t* test, value > 50/chance, # = *P* < 0.05, ## = *P* < 0.01 ### = *P* < 0.001). There were no differences between groups (*C3ar1^+/+^* versus *C3ar1^tm1Cge/tm1Cge^* Student’s *t* test *t*_[37]_ = 0.42, *P* = 0.67). (**A–I**). Experimental units are individual mice. Cohort 1: *C3ar1^tm1Cge/tm1Cge^ N* = 34 (18 male, 16 female), *C3ar1^+/+^ N* = 35 (17 male, 18 female), except for EPM where one *C3ar1^tm1Cge/tm1Cge^* mouse (male) and two *C3ar1^+/+^* mice (one male, one female) were removed from analysis due to falling off the open arm. Cohort 2: *C3ar1^tm1Cge/tm1Cge^ N* = 20, *C3ar1^+/+^ N* = 19 (males and females combined), except for EPM where two female *C3ar1^tm1Cge/tm1Cge^* mice and one male *C3ar1^+/+^* mouse was removed from analysis. Data are expressed as mean ± 95% CI except for (**A**).

In Cohort 1, no significant genotype effects were observed for anxiety-like behaviours (Supplemental table 4 for description of anxiety-like metric selection) or locomotor activity. Specifically, there were no differences between genotypes in OF distance travelled (Fig. 7A), time spent in the centre 70% of the arena (Fig. 7B), or time in the periphery (Fig. 7C). Similarly, EPM measures—including time spent in open arms (Fig. 7D), in the middle (Fig. 7E), or in closed arms (Fig. 7F)—showed no genotype differences. All corresponding statistical results are provided in Supplemental table 5. To rule out any potential confounding effects from co-housing littermate mutants and wild-types,^82^ we also examined whether the number of *C3ar1*-deficient cage-mates influenced wild-type behaviour but found no consistent pattern or evidence of systematic anxiety-like effects in wild-types (Supplemental Fig. 12). To assess multiple measures of anxiety-like behaviour and locomotion along one another (Fig. 7H–I), we calculated z-scores for each behavioural outcome measure for *C3ar1*-deficient mice relative to wild-type controls (Table 3 for untransformed means).

**Table 3.** Mean behavioural outcome measures ± 95% CI in males and females of both genotypes

Age	Cohort 1				Cohort 2			
*C3ar1*status	+/+	-/-	+/+	-/-	+/+	-/-	+/+	-/-
Sex	F		M		F		M	
**Anxiety-related**	**Untransformed means**							
OF duration core (s)	25.92 ± 12.12	18.74 ± 4.60	19.81 ± 5.35	22.99 ± 7.73	25.06 ± 10.65	29.96 ± 24.36	32.19 ± 18.93	16.71 ± 3.87
OF duration centre (s)	106.94 ± 18.40	102.01 ± 16.36	97.18 ± 13.68	109.40 ± 17.99	105.42 ± 40.78	120.94 ± 39.65	133.80 ± 31.20	95.62 ± 13.10
OF duration periphery (s)	493.13 ± 18.41	498.07 ± 16.36	502.90 ± 13.68	490.66 ± 17.98	493.16 ± 42.58	478.89 ± 39.60	466.11 ± 31.25	504.15 ± 13.10
EPM duration open (s)	29.52 ± 11.52	28.92 ± 12.57	28.06 ± 12.03	28.19 ± 14.72	18.87 ± 8.77	20.45 ± 16.56	15.13 ± 7.96	12.15 ± 7.29
OF latency core (s)	30.66 ± 17.23	44.47 ± 34.34	29.89 ± 16.18	40.95 ± 19.45	34.19 ± 36.91	24.15 ± 20.50	25.86 ± 25.45	29.44 ± 52.19
OF latency centre (s)	10.02 ± 3.52	13.26 ± 8.07	12.35 ± 7.40	11.97 ± 4.95	5.79 ± 6.00	7.45 ± 7.79	5.22 ± 5.01	5.28 ± 4.26
EPM duration middle (s)	109.53 ± 17.89	102.17 ± 19.15	112.33 ± 24.24	122.79 ± 17.27	85.38 ± 21.11	72.59 ± 29.02	71.13 ± 14.54	74.93 ± 17.85
EPM duration closed (s)	176.11 ± 22.18	198.69 ± 19.25	188.78 ± 24.17	178.29 ± 17.22	214.24 ± 21.04	207.31 ± 38.82	227.88 ± 14.86	224.17 ± 17.79
EPM bouts open (#)	4.65 ± 1.92	3.19 ± 1.66	3.44 ± 1.33	2.94 ± 1.18	2.62 ± 1.48	3.43 ± 2.77	2.30 ± 1.12	1.91 ± 1.49
EPM head dips (#)	21.53 ± 6.20	18.12 ± 5.17	19.62 ± 6.29	22.31 ± 5.36	16.75 ± 4.55	17.57 ± 13.16	11.90 ± 3.14	16.73 ± 8.52
EPM stretch-attend postures (#)	19.00 ± 4.15	21.81 ± 2.98	23.62 ± 3.14	23.38 ± 2.87	16.62 ± 6.16	9.43 ± 6.04	13.50 ± 2.68	14.73 ± 4.10
EPM latency open (s)	65.31 ± 27.63	106.76 ± 53.38	92.95 ± 48.81	117.27 ± 51.75	75.66 ± 81.38	83.26 ± 103.55	95.40 ± 71.29	137.52 ± 70.66
**Other**								
PPI 3 dB (%)					21.11 ± 13.2	15.31 ± 6.40	22.87 ± 7.41	17.14 ± 7.70
PPI 6 dB (%)					40.63 ± 10.16	26.36 ± 8.74	39.49 ± 11.24	34.37 ± 10.65
PPI 12 dB (%)					56.81 ± 13.18	49.81 ± 13.28	57.07 ± 9.51	60.40 ± 8.38
Acoustic startle response at 120 dB (A.U.)					876.35 ± 432.36	1068.32 ± 284.31	1472.99 ± 360.84	1908.79 ± 655.66
NOR recognition index (%)					62.49 ± 11.20	62.60 ± 9.77	65.02 ± 7.66	68.16 ± 8.87
NOR total exploration training (s)					39.45 ± 15.62	38.58 ± 10.66	38.50 ± 12.16	41.12 ± 11.07
NOR total exploration test (s)					31.68 ± 10.07	32.41 ± 6.83	30.81 ± 9.17	33.58 ± 5.39

OF = open field, EPM = elevated plus maze, PPI = prepulse inhibition, NOR = novel object recognition

In Cohort 2, most anxiety-related and locomotion measures showed no genotype differences (Supplemental table 6), except for reduced distance travelled in the centre of the OF arena by *C3ar1*-deficient mice (uncorrected two-sample *t*-test *P* < 0.01, Cohen's *d* = 0.92). However, no genotype effect on distance was detected in the core of the OF arena or in the centre and core of the OF arena in Cohort 1.

The sample size (*N* = 38) of Cohort 2 was too small to reliably test for interactions between sex and genotype, meaning we only had enough statistical power to detect very large effects (Cohen’s *f* = 0.5 at alpha = 0.05 and 80% power), which were clearly not observed across behavioural measures. No sex-by-genotype interactions were observed in Cohort 1 (Supplemental table 5).

Additionally, PPI testing (performed exclusively on Cohort 2) showed no effect of *C3ar1* deletion, though PPI increased with prepulse intensity as expected (Fig. 7G), confirming that the test was set up to reliably measure this phenomenon. NOR testing (also performed exclusively on Cohort 2) revealed no genotype differences, with all groups demonstrating successful learning based on recognition indices significantly above chance levels (Fig. 7J).

Overall, these findings suggest that *C3ar1* deficiency does not result in robust anxiety-like or hyperactive phenotypes, nor deficits in recognition memory or PPI.

## Discussion

Here we used longitudinal neuroimaging and behavioural testing to investigate the impacts of *C3ar1* deletion on brain structure and function across early life development in mice. We found no robust evidence that *C3ar1* deficiency affects total or regional brain volume, white matter FA, global FC, global efficiency, clustering coefficient, or specific functional networks.

We also found no altered behavioural phenotype in either male or female *C3ar1*-deficient mice in adulthood. These results raise questions regarding the source of discrepancies between these data, our previous behavioural work^28,30^ and that of others,^19,29,31,33^ which may be attributed to our use of littermate controls in this study, as will be elaborated upon in the following sections. Overall, the lack of a behavioural phenotype combined with the lack of genotype effects on brain structure across modalities and time-points means that the role of C3aR1 in neural development and behaviour is likely context dependent.

### Absence of *C3ar1*-dependent effects on brain structure

Our morphometric analysis showed that total and regional brain volumes were not changed between *C3ar1*-deficient and wild-type mice. We were, however, able to detect sex-specific neurodevelopmental changes that have previously been reported in rodents, such as brain growth occurring later in females, minimal total and regional brain volume differences in adulthood and larger volume of MEPO and BNST in males,^61-63,83,84^ supporting the sensitivity of our methodology. Together with the low variability in our sample, our ability to detect these known sex differences means that we were well-positioned to identify potential genotype effects had they been present. These findings are also consistent with another recent longitudinal MRI study in *C3ar1* knockout mice, which reported no differences in neocortex or hippocampus volumes at three months of age.^85^

### Absence of *C3ar1*-dependent effects on white matter fractional anisotropy

In the brain, *C3ar1* is predominantly expressed by microglia,^86,87^ where it may influence their reactivity and phagocytosis.^21,22,24-26^ Notably, changes in microglial characteristics have been linked to developmental alterations in fractional anisotropy in both mice and humans.^40,41^ We found no *C3ar1*-dependent changes in the white matter despite showing the overall effect of time on fractional anisotropy, which is known to increase during development between adolescence and adulthood in both humans and mice.^88-92^ Lack of a phenotype after the deletion of a predominantly microglial gene may not be surprising since mice that completely lack microglia for their entire lifespan (achieved by deleting the super enhancer for macrophage colony-stimulating factor receptor, *Csf1r*) do not have overt neurodevelopmental phenotypes,^93^ and only show vulnerability in a pathological context involving neuroinflammation.^94^ This raises the possibility worth discussing that neurodevelopmental phenotypes associated with *C3ar1* deficiency may likewise only become apparent under neuroinflammatory conditions. There is, however, an important distinction to be made here between an immune trigger during development and an inflammatory stimulus in adult animals. A growing body of literature suggests that *C3ar1* deficiency tends to be mildly anti-inflammatory and protective in contexts such as chronic stress or other neuroinflammatory conditions^22,31,33^—a profile that opposes the proposed hypothesis that *C3ar1* deficiency leads to aberrant phenotypes when combined with an inflammatory insult. Additionally, it is apparently important to distinguish between chronic and acute inflammatory stimuli. For instance, a recent study using scRNA-Seq data from the hippocampus of *C3ar1*-deficient mice given an acute lipopolysaccharide challenge (1 mg/kg i.p.) found that astrocyte, microglia, and oligodendrocyte activation signatures were completely *C3ar1*-independent,^85^ indicating that an acute systemic stimulus may be insufficient to reveal a requirement for *C3ar1* in the brain.

With this in mind, it is difficult to support a scenario in which the presence of *C3ar1* is beneficial during development but only under neuroinflammatory conditions, given that available data suggest it has either the opposite effect or no effect in adulthood, and there are currently no littermate-controlled studies to substantiate this developmental hypothesis. However, our study cannot definitively exclude this possibility. Future studies could address this question by incorporating maternal immune activation models or by testing *C3ar1*-deficiency in sensitized backgrounds, such as in the presence of *C4A* overexpression—a known genetic risk factor for schizophrenia^6^ that also affects white matter integrity.^95^

### Functional connectivity appears *C3ar1*-independent

We did not detect genotype-dependent global FC changes nor changes to clustering coefficient and global efficiency at either time-point. We did, however, observe a genotype-independent developmental increase in global efficiency and clustering coefficient with time, which has been reported before^73,89,96^ particularly at lower sparsities,^97^ suggesting that our method was sensitive enough to pick up developmentally relevant effects. Compared to previous microglia gene knockout neuroimaging studies,^66-68^ which have used smaller samples (7–20 mice per group) and detected large effects (Cohen’s *d* = 0.85–2.2),^66-68^ our sample size was substantially larger (34 mice per group with sexes combined) and provided 80% power to detect up to moderate effects (Cohen’s *d* = 0.7). However, we acknowledge that smaller genotype effects below these thresholds may not have been detected, and thus the possibility of Type II error for subtle differences cannot be excluded.

Similarly to whole brain analyses, we did not observe any genotype-dependent changes to specific brain networks, including anxiety networks (Fig. 5), which we hypothesized to be affected based on our previous behavioural results.^30^ However, our anxiety network connectivity null findings are internally consistent with the absence of an anxiety-like phenotype in the cohorts tested in this study. Our data combined with another recent null report for an anxiety-like effect in the EPM in *C3ar1*-deficient animals^33^ suggests that *C3ar1* deficiency alone is not enough to result in anxiety-like behaviour or changes to anxiety networks.

Although we did not detect network-specific effects, *C3ar1*-deficient mice exhibited subtle but widespread increases in resting-state FC in voxel-wise seed-based analyses during adolescence across nearly all seeds examined, with a weaker effect persisting into adulthood (Fig. 6 and Supplemental fig. 9–11). These increases in connectivity may be noise but they could reflect developmental alterations in circuit properties caused by the absence of C3aR1 that are not conclusively detectable by MRI. For now, we urge caution with this latter interpretation for two reasons that should be considered together. These connectivity changes were isolated findings—that is, they were not associated with any behavioural changes, nor were they accompanied by any structural alterations. This would be unexpected if a larger number of neurons were present due to, for example, a deficit in perinatal neuron phagocytosis as reported for *Cr3* knockouts.^68^ Second, the voxel-wise seed-based analysis is the least robust presented here, as the observed changes were largely transient, weak, not confined to a specific subnetwork, and not corrected for multiple comparisons across seeds. Future studies investigating FC in *C3ar1*-deficient mice should incorporate repeated measures from the same individual during adolescence to improve robustness as well as assessing synaptic transmission more directly, for example by electrophysiology in brain slices, although it is still unclear which brain regions should be targeted with the latter approach.

### Behavioural outcomes were unaffected by *C3ar1* deficiency

Neither anxiety-like behaviours nor locomotor activity showed significant differences between genotypes across multiple testing paradigms, apart from a small decrease in ambulation of *C3ar1*-deficient mice in the aversive central zone of the OF arena, which in the absence of a locomotor phenotype could index anxiety-like behaviour.^98^ This latter change, however, was not reproducible across study cohorts and was no longer observed in the same study in the core of the OF arena. Tests for recognition memory and sensorimotor gating, NOR and PPI, also yielded null results.

Unlike previous studies of *C3ar1*-deficient mice, our study used littermate control animals, meaning that wild-type mice were co-housed with their *C3ar1*-deficient siblings. This approach minimizes potential environmental differences between groups, which is especially important in neurobehavioural studies where subtle environmental factors can significantly affect outcomes. While the use of littermate controls is widely regarded as best practice in such contexts,^37,99,100^ it is not without its own potential confounds. For instance, abnormal behaviours exhibited by genetically altered animals can influence the behaviour of co-housed wild-type mice, particularly when the phenotype is pronounced, such as in cases of increased aggression.^82^ However, as aggression has not been reported in *C3ar1*-deficient mice, and their previously described behavioural phenotype was not notably severe, we considered such an influence unlikely in our study, especially as the anxiety-like behaviour of our wild-type mice did not seem to depend on the number of *C3ar1*-deficient cage-mates (Supplemental Fig. 12).

The complement system plays an essential role in normal pregnancy and parturition.^101^ A notable aspect of using a littermate design is that mixed-genotype litters are produced by breeding heterozygous parents, whereas in most previous studies of *C3ar1* deficiency, both the mother and offspring were homozygous knockouts. An exception is a report of hyperactivity in *C3ar1* knockout mice in which the knockouts were generated from heterozygous incrosses.^29^ This raises the possibility that some behavioural effects in *C3ar1*-deficient mice (anxiety-like behaviour and a memory deficit) may have resulted from altered intrauterine environments or care by *C3ar1*-deficient mothers. If this is indeed the reason for the previously observed adult behavioural phenotypes, two scenarios should be considered. In the first, unlikely scenario, the *C3ar1*-deficient offspring are *uniquely sensitive* to C3aR1-dependent *in utero* conditions or maternal care deficits. In the second scenario, the adult phenotypes are not specific to *C3ar1*-deficiency meaning that wild-type mice would have been similarly affected. These scenarios can be tested by using pup transfer and *in-vitro* fertilization experiments. For now, we were able to avoid measuring these non-specific effects on behaviour by using a littermate design.

The genetic background of mutants is another factor that varies between laboratories and is known to affect phenotypic expression. For example, heterozygous knockout of the autism-associated gene *CHD8* has varying effects in 33 sub-strains,^102^ mirroring heterogeneity observed in human *CHD8* haploinsufficiency. While that study identified significant variability across sub-strains, these profiles consistently differed from that of wild-type littermate controls. In contrast, our study found no robust genotype-dependent differences in the brain globally and across measures or in behaviour, apart from a single internally non-reproducible behavioural outcome measure, meaning that the effect of C3aR1 on behaviour would have to be entirely dependent on modifying genes or environmental factors.

Masking of a knockout phenotype can also occur through genetic compensation—an incompletely understood molecular process that leads to the upregulation of nearby and related genes, possibly through mutant RNA decay.^103^ To rule this out, scRNA-Seq analysis would have to be conducted at every time-point and tissue of interest. For now, it is reassuring that in adult *C3ar1^tm1Cge/tm1Cge^* hippocampus bulk RNASeq data, GPCRs do not appear to be upregulated at baseline.^104^

Finally, genetic drift could have precluded reproducibility of previously reported phenotypes. Mice accumulate spontaneous mutations that rapidly reach homozygosity within small colonies, speeding up drift, a phenomenon recognized as early as the 1980s.^34^ So far, our current work is the first to study behaviour in *C3ar1* deficient mice using a littermate design, which minimizes the number of genetic loci that differ between mutant and control, all of which could influence measured phenotypes.

### Limitations

We provided high-level global data on *C3ar1*-deficient mice. While our study had notable strengths, including the use of a littermate design and the inclusion of female animals, it is not an exhaustive characterization of the mutant.

While we measured brain region volumes, we cannot definitively address more reductionist questions, such as cellular composition which would have required single cell RNASeq or antibody staining and microscopy. Similarly, for axonal integrity analysis for which we used the proxy of dMRI-derived fractional anisotropy, the gold standard would have been electron microscopy. However, a more granular approach would have necessitated a trade-off with throughput—something that is hard to justify in the absence of strong hypotheses regarding the developmental expression pattern of *C3ar1*. Additionally, our fMRI analysis relied on a specific parcellation of the brain consisting of 36 regions, and different parcellations could yield varying results.^105,106^ We encourage others exploring our datasets to therefore experiment with alternative parcellations.

It is important to note that rodent fMRI is typically performed under anaesthesia. We used a previously validated combined medetomidine and isoflurane anaesthesia selected for preserving maximum number of functional connections.^50^ Nevertheless, anaesthesia can influence FC patterns. Medetomidine, in particular, has been reported to reduce inter-hemispheric FC,^107^ and the combination with isoflurane may further alter connectivity signatures, particularly as it can stimulate the immune system^108^ and therefore may hypothetically interact with the complement system. While there is no evidence that this protocol differentially affects wild-type and *C3ar1* knockout animals, it cannot be ruled out that *C3ar1* deficiency affects anaesthesia response in unforeseen ways, although this would be a more pressing concern in the presence of a strong *C3ar1*-dependent phenotype. It is also possible that our anaesthesia protocol may have masked a genotype effect. However, the absence of a consistent trend across measures supports the interpretation that this is a true null result.

### Future directions

Aside from the role of C3aR1 in brain structure and function, many aspects of the basic biology of C3aR1 remain unclear, including its expression pattern in the healthy brain, with little information available on the cell types where it is expressed, its expression contexts and time-points. It is also unclear which G-proteins C3aR1 signals through in different brain cell types. Future studies could integrate toolkits like TRUPATH, a suite of Gαβγ biosensors for analysing G-protein coupling preferences,^109^ with transcriptomics to address these questions. This approach would be particularly valuable, as it could enable the use of chemogenetics to activate the same G-protein as C3aR1 in specific cell types and contexts to study its function. Only after these fundamental investigations have been completed should *C3ar1* deficiency be examined at the behavioural level—particularly following immune challenges known to activate complement in the brain during critical periods of development.

## Supplementary Material

fcaf422_Supplementary_Data

## Data Availability

Behavioural videos have been deposited via Zenodo. Due to repository size limitations, datasets were divided by both experimental cohort and behavioural task; https://doi.org/10.5281/zenodo.17100928 (Cohort 1 open field), https://doi.org/10.5281/zenodo.17107902 (Cohort 1 elevated plus maze), https://doi.org/10.5281/zenodo.17113481 (Cohort 2 open field and elevated plus maze) and https://doi.org/10.5281/zenodo.17113949 (Cohort 2 novel object recognition training and test videos). Raw MRI images and relevant metadata can be found on OpenNeuro: https://openneuro.org/datasets/ds006663/versions/1.0.1 (Cohort 1, doi:10.18112/openneuro.ds006663.v1.0.1) https://openneuro.org/datasets/ds006670/versions/1.0.0 (Cohort 2, doi:10.18112/openneuro.ds006670.v1.0.0) Intermediary analysis files, including preprocessed data, are maintained alongside the code at https://github.com/hannalemmik/C3ar1_longitudinal_MRI_paper_2025. The analysis code used to reproduce all figures in this manuscript is available at https://github.com/hannalemmik/C3ar1_longitudinal_MRI_paper_2025. Additional MRI processing scripts are available upon request from Eugene Kim.
