# Supplementary material for: Complement receptor C3ar1 deficiency does not alter brain structure or functional connectivity across early life development
Source: Brain Commun. 2025 Oct 28;7(6):fcaf422. doi: 10.1093/braincomms/fcaf422 (PMC12646079; doi:10.1093/braincomms/fcaf422)
Supplement: fcaf422_Supplementary_Data [file fcaf422_supplementary_data.pdf]

Supplemental methods

Further notes on experimental animals

Experimental animals (*C3ar1*$^{tm1Cge/tm1Cge}$ and *C3ar1*$^{+/+}$ littermates) were generated through heterozygote incrosses. The resulting genotype distribution followed Mendelian ratios, with a non-significant trend toward an increased proportion of *C3ar1*$^{+/+}$ animals (**Supplemental figure 1A**). This trend cannot be fully explained by early deaths ($N = 3$), which may be attributable to maternal cannibalism. Although the observed bias was not statistically significant, it remains possible that *C3ar1* deficiency (heterozygous or homozygous) may be detrimental *in utero*. Future studies with larger sample sizes will be necessary to clarify this possibility.

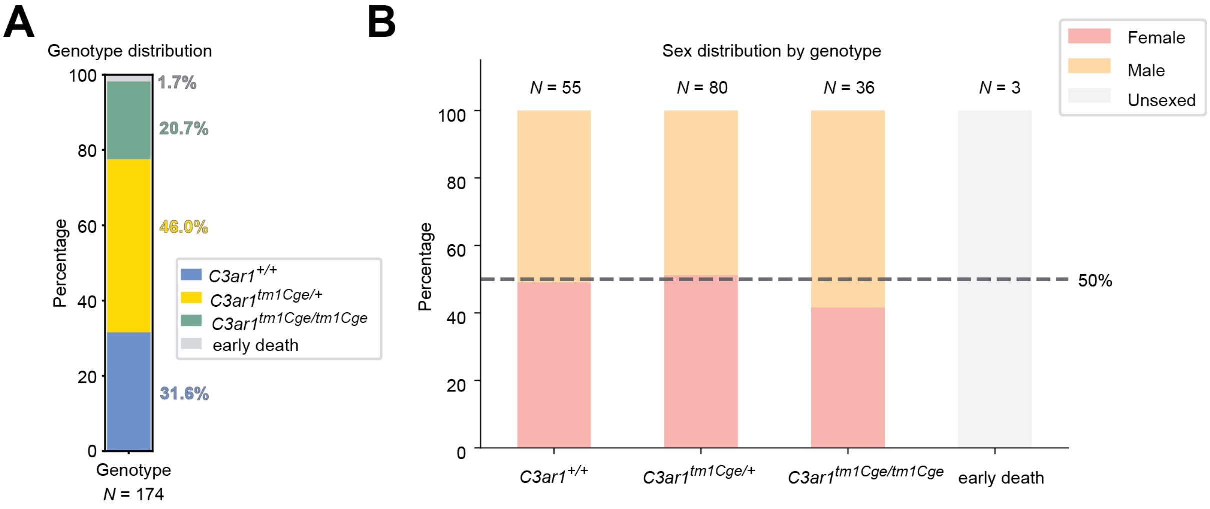

Supplemental figure 1 | Genotype and sex ratios. (**A**) Genotype ratios for litters in Cohort 1 and 2. Litters that contained animals with an unsuccessful genotyping result were removed due to ambiguity. χ^2 statistic against expected Mendelian ratios (25%, 50%, 25%): 4.93, *p*-value: 0.09. (**B**) Sex ratios for Cohort 1 and 2. Only *C3ar1*$^{tm1Cge/tm1Cge}$ mice appeared to show a slightly biased sex ratio but this was not significant (χ^2 statistic: 1.0, *p*-value: 0.3). (**A–B**) Early deaths refer to animals who died before the first 2 weeks.

The heterozygote breeders were generated by outcrossing heterozygous mice to bought wild-type Charles River C57BL/6Js. The breeders used to produce the experimental animals were derived either from the first, second or third of these outcrosses. Sibling crosses were not conducted, and parental age was between 2–4 months to minimise genetic drift. Charles River C57BL/6Js are on Jackson's laboratory genetic stability program, which is designed to slow down genetic drift by introducing breeders from cryopreserved stocks into their colonies every 12 generations.

Experimental mice were housed in individually ventilated cages under controlled temperature (20–25°C), humidity (50–60%), and a 12-hour light-dark cycle (lights on at 7:00 AM, lights off at 7:00 PM). Environmental enrichment included nesting materials, tunnels, and chew sticks. Mice had *ad libitum* access to irradiated rodent chow and autoclaved water. Animals were group-housed (2–4 mice per cage), with males and females housed separately after weaning (PND21±2). Genotyping was conducted on ear biopsy DNA by Transnetyx using probes targeting the neomycin cassette for the mutant allele and intron 1 for the wild-type allele. No mismatches were identified through double-genotyping 20% of the study cohorts.

The colony of *C3ar1*^{tm1Cge} mice was monitored routinely by both the experimenter and KCL Biological Services staff. No health or behavioural abnormalities were observed during routine husbandry or experimental procedures. Predefined humane endpoints were established in accordance with institutional welfare guidelines, which included criteria such as 20% body weight loss, reduced activity, coat condition, and signs of pain or distress. Throughout the study period, no mice met any listed criteria for early humane intervention.

A comprehensive research and analysis plan was developed for this study. The study rationale and statistical procedures were evaluated and approved by Hanna Lemmik's thesis committee and supervisors. Ethical considerations for individual experiments were formally reviewed by the KCL Biological Services Unit, in accordance with institutional and regulatory guidelines.

RNA extraction, cDNA synthesis, gel electrophoresis

RNA was extracted using the ReliaPrep™ miRNA Cell and Tissue Miniprep System (Promega, #Z6211) according to the manufacturer's instructions. RNA concentration was determined with a NanoDrop spectrophotometer (Thermo Scientific, NanoDrop 2000), yielding values between 25.5 ng/µl and 227.5 ng/µl. Reverse transcription PCR (RT-PCR) was performed using the LunaScript® RT SuperMix Kit (NEB, #E3010L), following the manufacturer's instructions. Depending on RNA yield, either 100 or 500 ng of RNA was used per reaction.

For cDNA PCR and gel electrophoresis, we used GoTaq® G2 Master Mix (NEB, #M7822). We amplified a 79 basepair (bp) fragment in the deleted region alongside a 372 bp region in the *C3ar1* cDNA that was located outside the deleted region and downstream of an alternative start codon identified through Benchling. *Gapdh* primers were included in each reaction to confirm amplification efficiency. PCR products were analysed on a 2% agarose gel stained with GelRed. For PCR primers, reaction composition and cycling conditions see **Supplemental methods tables 1–3**).

qPCR and primer sequences

For qPCR, we amplified the previously mentioned 79 bp fragment in the deleted region. Samples were analysed in triplicate on 96-well plates (Applied Biosystems, #4346906) with Luna® Universal qPCR Master Mix (NEB, #M3003L), and readings were obtained using an Applied Biosystems StepOnePlus plate reader. The amplification data were processed with the ΔΔCt method, normalising against the housekeeping gene Hypoxanthine phosphoribosyltransferase 1 (Hprt). Genotypes were arranged alternately across the plate to minimise bias. To verify M2-like polarisation, the expression of M2-specific markers Arginase 1 (*Arg1*) and the mannose receptor Cluster of differentiation 206 (*Cd206*) were assessed. For qPCR primers and cycling conditions, see Supplemental methods table 1 and 3 respectively.

Supplemental methods table 1 Primer sequences

Primer name	Forward	Reverse	Application
C3ar1	AGGATTTGTTGGTGGCTCGCA	CTCCATGGCTCAGTCAAGCACA	PCR and qPCR deleted region
C3ar1	GCTTCCTGGTGCCGTTTTTC	AGTTGGTAGAGTGCGTGAGC	PCR of putative alternative transcript (3' end of exon 2 after alternative start codon)
Hprt	AGTCCCAGCGTCGTGATTAGCG	TTGAGCACACAGAGGGCCACAA	qPCR housekeeping gene
Arg1	GGCTTGCGAGACGTAGACCC	GTCCAGCCCGTCGACATCAAA	qPCR verification of M2 polarisation
Cd206	CCGGAGGGTGCAGACAAAGG	TCGTCCACAGTCCACCGAAAC	qPCR verification of M2 polarisation
Gapdh	CCTAGACAAAATGGTGAA	GACTCCACGACATACTCAGC	PCR amplification efficiency control

Supplemental methods table 2 PCR reaction

Amount (µl)	Reagent
12.5	GoTaq® G2 Master Mix (Green)
1	*C3ar1* forward primer, 10 mM
1	*C3ar1* reverse primer, 10 mM
1	*Gapdh* forward primer, 10 mM
1	*Gapdh* reverse primer, 10 mM
7.5	H_2O
1	Template DNA
**Final volume: 25 µl**	

Supplemental methods table 3 | Cycling conditions

Step	Time	Temperature
1. Initial denaturation	5 min	94°C
2. Denaturation	30 sec	94°C
3. Annealing	30 sec	60°C
4. Extension	1 min	72°C
5. Repeat steps 2-4	Repeat 35 x	
6. Final extension	5 min	72°C

MRI acquisition parameters

For *in vivo* imaging, we first acquired an Actual Flip Angle Imaging (AFI) sequence for B1 mapping. Then we acquired three types of 3D multi-gradient-echo images that were used for creating study-specific templates: magnetization-transfer weighted (MTw), proton-density weighted (PDw), and T1-weighted (T1w). Subsequently, T2-weighted Rapid Acquisition with Relaxation Enhancement (RARE) images were obtained. Diffusion weighted images (DWI) were then acquired using a single-shot spin-echo echo planar imaging (EPI) sequence. Finally, for the longitudinal magnetic resonance imaging (MRI) study, blood oxygen level dependant (BOLD) resting state functional (rsf)MRI data were acquired using a single-shot gradient-echo echo planar imaging (EPI) sequence with 720 repetitions. Additionally, spin-echo EPI image pairs with opposing phase-encoding polarity were recorded to enable correction of susceptibility-induced distortions. The *in vivo* scanning session lasted 1–1.5 hours. Further acquisition parameters are listed in **Supplemental methods table 4**.

Supplemental methods table 4 MRI acquisition parameters

Image type	TR (ms)	TE (ms)	Flip angle (°)	Aver-ages	Bandwidth (kHz)	FoV	Matrix	Other
AFI	20/ 100	2.85	55	1	25	16.2 x 16.2 x 9	42 x 42 x 24	
MTw	24	2.5	6	2	100	16.2 x 16.2 x 9	108 × 108 × 60	6 echoes with 2.1 ms spacing; MT pulse: gaussian, 4 ms, amplitude 10 μT, offset -3 kHz, bandwidth 685 Hz
PDw	20	2.5	4	2	100	16.2 x 16.2 x 9	108 × 108 × 60	7 echoes with 2.1 ms spacing
T1w	20	2.5	20	2	100	16.2 x 16.2 x 9	108 × 108 × 60	7 echoes with 2.1 ms spacing
T2w *in vivo*	5000	42	90/ 180	4	50	16 x 12	128 × 96	32 slices, slice thickness 0.5, RARE factor 8
DWI *in vivo*	3000	21	90/ 180	2	357	19.2 x12	96 x 60	Single shot spin-echo planar imaging, 30 slices, slice thickness 0.5 mm, three diffusion shells: b = 350 s/mm² with 9 directions, b = 1000 s/mm² with 34 directions, b = 2000 s/mm² with 78 directions (δ = 3 ms, Δ = 11 ms), 3 b0 images per shell
DWI *ex vivo*	300	28.5	90/ 180		300	25 × 24 × 18	200 × 192 × 144	Stejskal-Tanner pulsed gradient spin echo sequences with a 3D segmented EPI readout. 12 segments and a total of 90 diffusion-weighted images acquired at a b-value of 4000 s/mm² (δ = 4 ms, Δ = 13 ms)
BOLD fMRI	1000	15	55	1	200	20 x 20	64 x 64	Single-shot gradient-echo EPI sequence, 16 slices, slice thickness 0.5, 720 repetitions

FoV = field of view, AFI = Actual Flip Angle, TR = repetition time, TE = echo time, MTw = magnetization-transfer weighted, PDw = proton-density weighted, DWI = diffusion weighted images, EPI = echo planar imaging, RARE = rapid acquisition with relaxation enhancement, BOLD fMRI = blood oxygen-level dependent.

Structural MR image pre-processing

For preprocessing MTw, T1w, and PDw images, de-ringing was conducted using the MRtrix3's[1] `mrdegibbs` command. MTw, T1w, and PDw images were averaged across echo times, rigidly co-registered using Advanced Normalization Tools[2] `antsRegistration`, and used for template construction (see below).

For DWI, MRtrix3 `dwidenoise` was used for de-noising, MRtrix3 `mrdegibbs` for de-ringing, and FSL's[3,4] `topup` and `eddy` for susceptibility and eddy-current distortion and motion correction. FSL's `dtifit` was used for diffusion tensor imaging (DTI) model fitting, enabling the calculation of fractional anisotropy (FA), mean diffusivity (MD) and axial diffusivity (AD), the latter two which are not reported in this manuscript for brevity. For a more detailed diffusion processing protocol see Kim *et al.* 2023.[5]

BOLD fMRI pre-processing

Images were largely pre-processed using the Analysis of Functional NeuroImages (AFNI) toolkit. Slice timing correction was performed using the `3dTshift` package, despiking with `3dDespike`, and motion correction was applied with `3dvolreg`. The motion-corrected time average was then registered to each subject's own T2w image using ANTs, followed by registration to the study template

The images were also distortion corrected using FSL's `topup`, with the distortion estimated using an auxiliary pair of spin-echo images with opposite phase encoding polarity and otherwise matching acquisition parameters to the gradient echo EPI used for the BOLD signal. Prior to analysis, corrected images were band-pass filtered at 0.01–0.2 Hz with AFNI's `3dTproject` to remove low-frequency scanner drift noise and high-frequency physiological noise. Nuisance variables (movement and CSF signal) were also simultaneously regressed out of the signal at this stage. Finally, spatial smoothing was applied using AFNI's `3dBlurInMask` with a FWHM kernel.

Ex vivo MRI

Following the adulthood *in vivo* scan in Cohort 1, mice were perfused transcardially with 20 mL phosphate-buffered saline (PBS) followed by 4% paraformaldehyde (PFA). Heads were stored in PFA for 48 hours, then transferred to PBS containing 0.05% sodium azide and 2 mM gadolinium-based contrast agent (Gd-DO3A-butrol). Brains were scanned *in cranio* in groups of four using a custom-made holder immersed in perfluoropolyether (Galden®, Solvay).

For *ex vivo* morphometric analysis, 3D T2-weighted images were acquired using RARE sequences, with a total scan duration of 1 hour and 5 minutes. *Ex vivo* diffusion-weighted images were obtained using Stejskal-Tanner pulsed gradient spin-echo sequences with a 3D segmented EPI readout. Three b0 images were collected at the beginning of three blocks of 30 diffusion-weighted images. The total scan time for this acquisition was 14 hours and 15 minutes. Further acquisition parameter info can be found in Supplemental methods table 5

Supplemental methods table 5 *Ex vivo* MRI acquisition parameters

Image type	TR (ms)	TE (ms)	Flip angle (°)	Averages	Bandwidth (kHz)	FoV	Matrix	Other
T2w ex vivo	300	30	90/ 180	1	50	25 x 24 x 18	250 x 240 x 180	RARE factor 4, scan time = 1 h 5 min
DWI ex vivo	300	28.5	90/ 180		300	25 × 24 × 18	200 × 192 × 144	Stejskal-Tanner pulsed gradient spin echo sequences with a 3D segmented EPI readout. 12 segments and a total of 90 diffusion-weighted images acquired at a b-value of 4000 s/mm² (δ = 4 ms, Δ = 13 ms)

FoV = field of view, TR = repetition time, TE = echo time, DWI = diffusion weighted images, RARE = rapid acquisition with relaxation enhancement.

General notes on behavioural procedures

For Cohort 1, EPM was administered as the first test, followed by OF. For Cohort 2, OF was the first test, followed by NOR test after two days of low light habituation (4 lux), EPM, and PPI.

Handling of the mice began 2–3 days prior to the behavioural testing battery. By the start of the experiments, the mice sat comfortably on the experimenter's hand. Mice were handled using cardboard tunnels to minimise stress, and tail handling was avoided.

Behavioural tests were conducted during the dark phase (between 7:00 PM and 11:00 PM) of the light-dark cycle to align with the active period of mice. Mice were randomised by genotype and counter-balanced by sex, with the experimenter systematically blinded to genotype throughout testing and analysis though the allocation of a study ID and test order ID, respectively.

For cleaning of the test apparatus, we used 70% EtOH for all arenas except for the EPM arena for which we used Virusolve (Amity International) to avoid damage to the material. Behaviour was recorded using a Google Pixel 5a camera at 1080p/60 fps. Videos were then cropped and down-sampled using FFmpeg.

Open field test

Mice were placed in a 40×40×40 cm white arena and allowed to explore freely. For Cohort 1, dim red light (4 lux) was used, while for Cohort 2, the OF test was conducted under bright overhead lighting (500 lux). 10-minute videos were analysed using CleverSys (VA, USA). Outcome parameters included time spent in the centre, total distance travelled, velocity, and thigmotaxis (edge exploration).

Novel object recognition

Cohort 2 mice were initially habituated to the arena under low light conditions (4 lux) over two days, five minutes per session. On the training day, two identical objects were introduced, and mice were allowed to explore for five minutes. After a one-hour delay, one object was replaced with a novel one. The videos were analysed using CleverSys software stereotypic event "Sniffing" module. Objects were manually outlined with the polygon tool. An interaction with an object was recorded when the mouse's nose was within 5 mm of the object. The novelty preference was determined by calculating the proportion of time spent exploring the novel object relative to the total exploration time of both objects, with a recognition index chance level of 50%.

Elevated plus maze

For Cohort 1, EPM was administered to behaviourally naïve animals, while for Cohort 2, it was conducted after OF and NOR tests. In both cases, mice were placed in the closed arm of the arena (65×65×55 cm, elevated 40 cm) under full overhead lighting (500 lux) and allowed to explore for five minutes. The arena was divided into closed, middle, and open areas for analysis with CleverSys software. The number of head dips and stretch-attend postures was recorded using BORIS software, and testing accuracy was compared with the results of an independent scorer.

Prepulse inhibition

Like NOR, PPI was only conducted in Cohort 2. We used an acoustic startle chamber (SR-LAB, San Diego Instruments, San Diego, CA, USA) with a cylindrical Plexiglas enclosure horizontally mounted on a mobile platform within a sound-proofed isolation chamber. A high-frequency loudspeaker positioned above the enclosure emitted continuous background noise at 65 dB, along with the experimental acoustic stimuli. The startle response was recorded by converting Plexiglas enclosure vibrations into millivolt signals using a piezoelectric unit.

Each session started with a five-minute acclimatisation period to the 65 dB background noise, followed by five startle-alone trials at 120 dB to ensure habituation. Mice then received 10 prepulse stimuli at 3, 6, and 12 dB above background, each preceding a 120 dB pulse in a pseudo-randomised order, interspersed with 10 no-stimulus trials and 10 startle-alone trials. The session concluded with five final startle-alone pulses. The inter-trial interval was randomised between 9–15 seconds to prevent expectation-based modulation of the startle response. %PPI was calculated for each prepulse using the formula: $\%PPI = \frac{pulse\ alone\ (mV_{Max}) - pulse\ with\ prepulse\ (mV_{Max})}{pulse\ alone\ (mV_{Max})}$.

Exclusions

All MRI datasets were visually inspected for distortions and artifacts; images with severely compromised quality were excluded from further analysis. For example, several adult diffusion scans displayed darker contrast in the left quadrant due to equipment issues during acquisition, which would have compromised the analysis. In BOLD fMRI datasets, scans were further excluded if principal component analysis failed to reveal identifiable resting-state networks, indicating impaired functional connectivity. No animals were excluded from behavioural experiments except in the EPM analysis: some animals fell off the equipment while exploring the open arm, which may have altered their behavioural patterns, as they tended to remain in the closed arms, meaning that this may not have represented genuine behavioural measures for this test. Individual sample sizes for each outcome measure have been reported in the main text figure legends.

Supplemental figures and tables

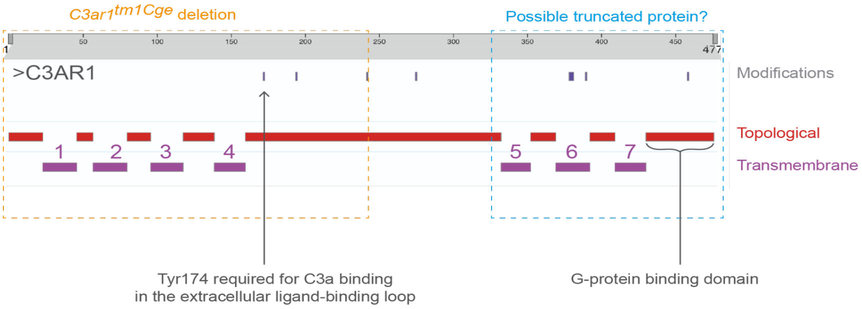

Supplemental figure 2 | Schematic of the C3AR1 protein. Marked are the *C3ar1*^{tm1Cge} deleted region (246 residues), removing the 4 transmembrane domains and the C3a binding residue at Tyr174. There is an alternative start codon 80 residues downstream of the mutation, meaning that a truncated protein that retains the three last transmembrane domains and the G-protein binding region is possible.

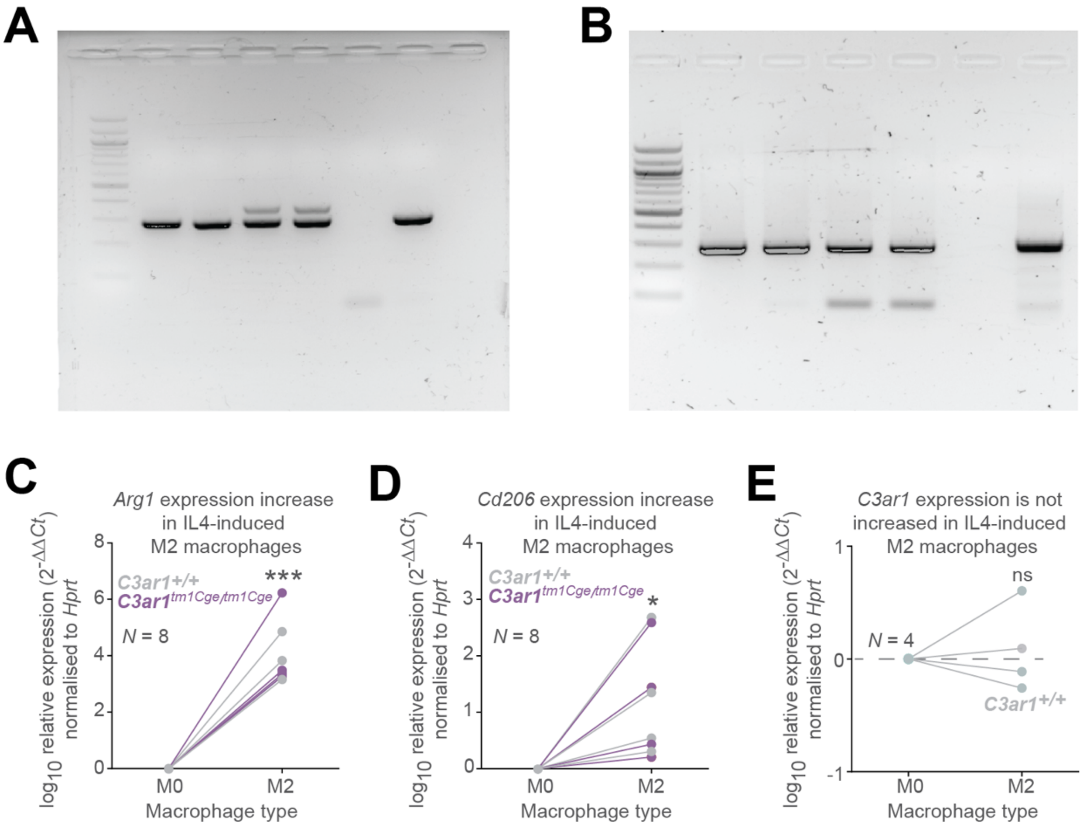

Supplemental figure 3 | *C3ar1*$^{tm1Cge/tm1Cge}$ M0 and M2 macrophages do not express *C3ar1*. Analysis of cDNA extracted from *C3ar1*$^{+/+}$ and *C3ar1*$^{tm1Cge/tm1Cge}$ bone marrow derived macrophages. (**A**–**B**) Uncropped gel images of the (**A**) hypothesized alternative *C3ar1* transcript corresponding to main text Figure 1B and (**B**) canonical transcript corresponding to Figure 1C. (**C**–**D**) qPCR of bone marrow derived macrophages. Adding recombinant interleukin 4 (IL4) to bone marrow derived M0-like macrophages increased the expression of M2-like macrophage markers (**C**) *Arg1* and (**D**) *Cd206* (one-sample *t* tests, M2 expression level difference from 0 (M0, baseline), *Arg1* $t_{[7]} = 10.29$, $p < 0.001$, *Cd206* $t_{[7]} = 3.37$, $p < 0.05$. N = 4 mice for each genotype. **(E)** No difference in *C3ar1* expression between M0 and M2 macrophages (one-sample *t* test, M2 expression level difference from 0 (M0, baseline), p = ns). (**C**–**E**) $N = 4$ mice for each genotype. *Arg1* = Arginase 1, *Cd206* = mannose receptor Cluster of Differentiation 206, *Hprt* = Hypoxanthine-Guanine Phosphoribosyltransferase 1 (housekeeping gene).

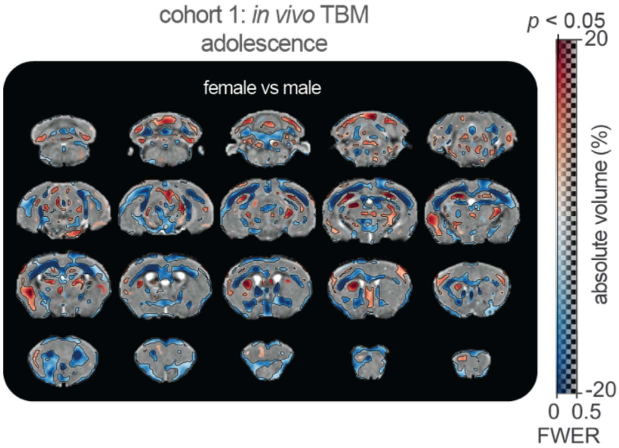

Supplemental figure 4 | Sex influences regional brain volumes in adulthood: Cohort 1 *in vivo* absolute volume data. Panel showing absolute regional volume changes (%) overlaid on study-specific coronal template (grey). Red hues signify areas larger in females and blue hues signify areas larger in males . Transparency of the colour overlay shows the statistical significance, ranging from FWE-corrected voxel-wise *t*-test *p* value 0.5 to 0 (transparent to opaque, respectively). Areas where FWE-corrected *p* value < 0.05 are demarcated with a black line, and where the *p* value > 0.5 are grey (no overlay). Genotypes are combined, males n = 35, 17 *C3ar1*$^{+/+}$ and 18 *C3ar1*$^{tm1Cge/tm1Cge}$; females n = 34, 18 *C3ar1*$^{+/+}$ and 16 *C3ar1*$^{tm1Cge/tm1Cge}$.

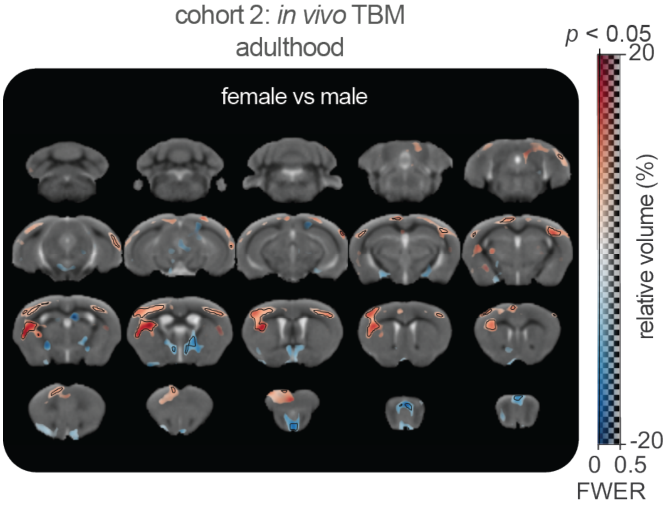

Supplemental figure 5 | Sex influences regional brain volumes in adulthood: Cohort 2 *in vivo* data**.** Panels showing relative brain volume changes (%) derived from TBM analysis of *in vivo* (FWE-corrected voxel-wise *t*-tests). Genotypes are combined, males n = 21, 11 *C3ar1*$^{+/+}$ and 10 *C3ar1*$^{tm1Cge/tm1Cge}$; females n = 16, 8 *C3ar1*$^{+/+}$ and 8 *C3ar1*$^{tm1Cge/tm1Cge}$.

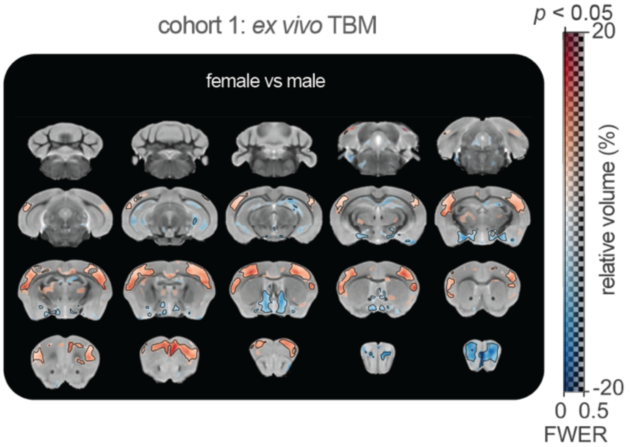

Supplemental figure 6 | Sex influences regional brain volumes in adulthood: Cohort 1 *ex vivo* regional volume data. Panels showing TBM relative brain volume changes (%), FWE-corrected voxel-wise *t*-tests. Genotypes are combined, males n = 35, 17 *C3ar1*$^{+/+}$ and 18 *C3ar1*$^{tm1Cge/tm1Cge}$; females n = 34, 18 *C3ar1*$^{+/+}$ and 16 *C3ar1*$^{tm1Cge/tm1Cge}$.

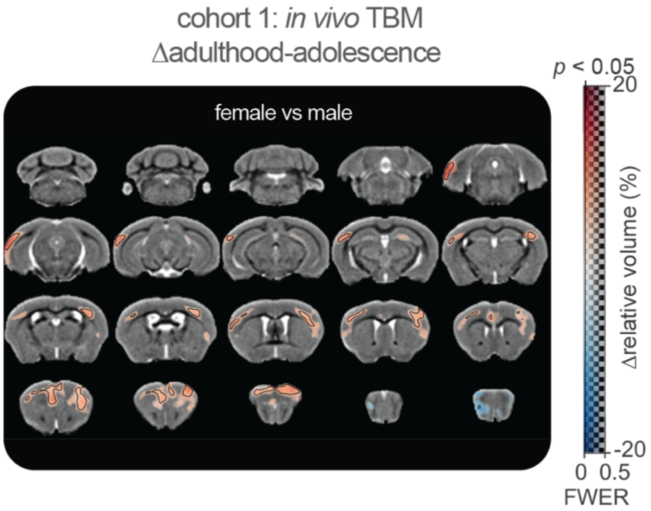

Supplemental figure 7 | Cortical area relative volume increased more in females than males between PND30 and PND90. Panels showing change in relative brain volume (Δadulthood – adolescence) between sexes, FWE-corrected voxel-wise *t*-tests. Genotypes are combined, males n = 35, 17 *C3ar1*$^{+/+}$ and 18 *C3ar1*$^{tm1Cge/tm1Cge}$; females n = 34, 18 *C3ar1*$^{+/+}$ and 16 *C3ar1*$^{tm1Cge/tm1Cge}$.

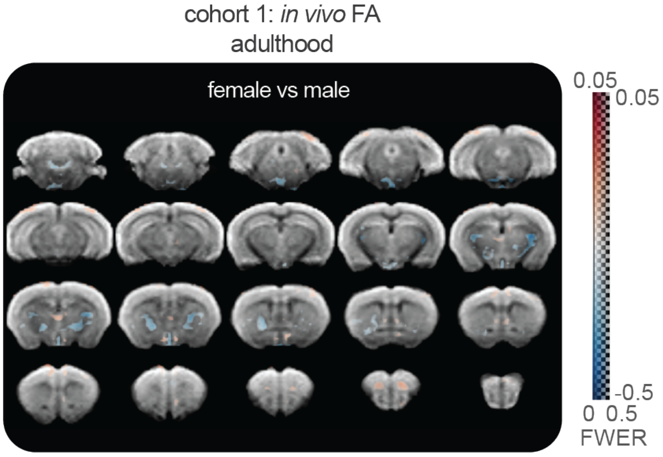

Supplemental figure 8 | Females show subthreshold decreases in FA at PND90 in voxel-wise fractional anisotropy analysis corrected for FWE. There are no significant voxels (no black contour) where sex effect is significant ($p < 0.05$), FWE-corrected voxel-wise *t*-tests. Genotypes are combined, males n = 35, 17 *C3ar1*$^{+/+}$ and 18 *C3ar1*$^{tm1Cge/tm1Cge}$; females n = 34, 18 *C3ar1*$^{+/+}$ and 16 *C3ar1*$^{tm1Cge/tm1Cge}$.

Supplemental table 1 Global functional connectivity metric *p* values with sexes combined corresponding to main text Figure 4.

Metric	*p*-value	Bonferroni *p*-value
FC PND30	0.1	0.3
FC PND90	0.32	0.96
FC change	0.64	1
GE PND30	0.02	0.06
GE PND90	0.1	0.28
GE change	0.66	1
CC PND30	0.1	0.3
CC PND90	0.2	0.61
CC change	0.71	1

C3ar1$^{tm1Cge/tm1Cge}$ vs *C3ar1*$^{+/+}$ permutation testing (10,000 iterations) uncorrected *p* values and Bonferroni connected *p*-values (n = 3 *p*-values per metric). FC = functional connectivity, CC = clustering coefficient, GE = global efficiency, PND = postnatal day, BH = Benjamini-Hochberg.

Supplemental table 2 Global functional connectivity metric *p* values with sexes separated corresponding to main text Figure 4.

Metric	Effect name	Standard error	*t*-value	Raw *p*-value	Corrected *p*-value
	Genotype	1.92	-1.07	0.286	1
FC PND30	Sex	0.93	2.2	0.028	0.763
	Interaction	2.78	-0.56	0.575	1
	Genotype	1.015	-1.85	0.064	1
FC PND90	Sex	1.85	1.4	0.161	1
	Interaction	2.64	0.06	0.948	1
	Genotype	0.79	0.35	0.727	1
FC change	Sex	3.36	0.05	0.956	1
	Interaction	3.86	0.54	0.588	1
	Genotype	0.50	-1.92	0.054	1
CC PND30	Sex	0.65	0.6	0.546	1
	Interaction	1.05	-0.36	0.721	1
	Genotype	0.93	-1.12	0.264	1
CC PND90	Sex	1.08	0.56	0.574	1
	Interaction	1.57	0.11	0.911	1
	Genotype	0.87	0.05	0.962	1
CC change	Sex	0.64	0.09	1	1
	Interaction	0.82	0.89	0.372	1
	Genotype	0.45	-1.82	0.069	1
GE PND30	Sex	0.34	0.45	0.653	1
	Interaction	0.61	-0.62	0.538	1
	Genotype	0.41	-2	0.046	1
GE PND90	Sex	0.36	2.16	0.031	0.834
	Interaction	0.78	0.03	0.976	1
	Genotype	0.72	0.15	0.881	1
GE change	Sex	0.54	1	0.317	1
	Interaction	1.07	0.42	0.672	1

Linear mixed-effects models with a random effect for subject to assess the effects of genotype, sex and their interaction with Bonferroni connected *p*-values (n = 3 *p*-values per metric). FC = functional connectivity, CC = clustering coefficient, GE = global efficiency, PND = postnatal day.

Supplemental table 3 Mean absolute anxiety-related region seed connectivity ANOVA *p* values with sexes separated.

Age	Effect name	DF1	DF2	MS	F	*p*-unc	np2
	Genotype	3	61	0.33	1.0	3.97E-01	0.05
PND90	Region	19	1159	0.78	174.65	1.48e-323	0.74
	Interaction	57	1159	0.01	1.33	5.37E-02	0.06
	Genotype	3	60	0.37	1.5	0.22	0.07
PND30	Region	19	1140	0.59	138.68	4.50E-280	0.7
	Interaction	57	1140	0.01	0.66	0.98	0.03

DF1 = degrees of freedom (numerator), DF2 = degrees of freedom (denominator), MS = mean squares (average variance explained), F = F statistic, *p*-unc = uncorrected *p* value, np2 = η_p^2 (partial eta squared effect size), PND = postnatal day.

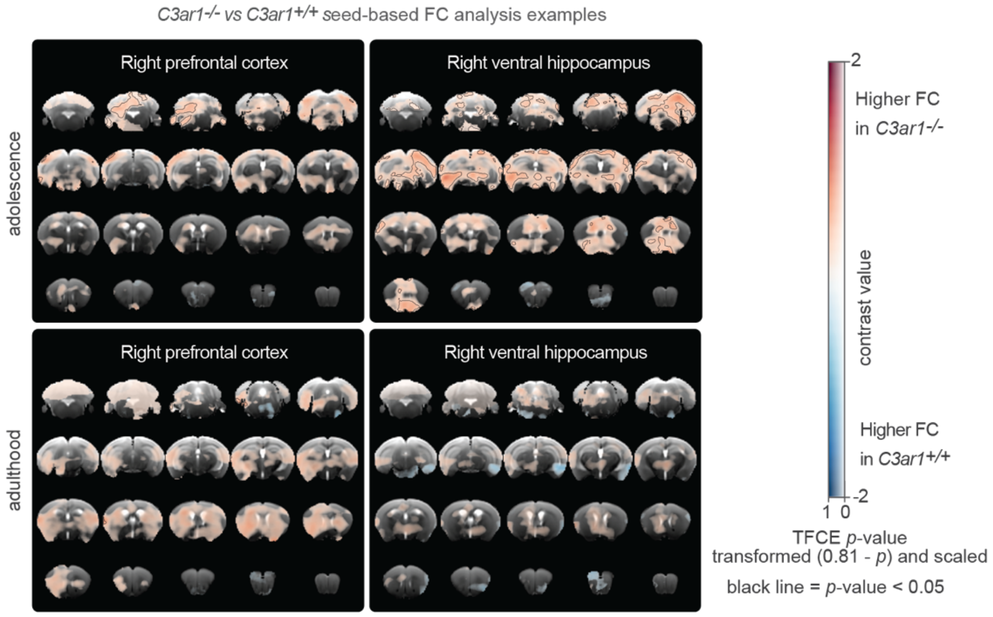

Supplemental figure 9 | Seed analysis examples in the right hemisphere. Examples of seed-based FC maps showing voxel-wise group differences between *C3ar1*$^{tm1Cge/tm1Cge}$ and *C3ar1*$^{+/+}$ mice (using *t* tests) with seeds placed in the right ventral hippocampus and right prefrontal cortex in adolescence and adulthood datasets. The dual scale bar displays contrast value on the x-axis and threshold free cluster enhancement (TFCE) *p*-values (transformed 0.81−p) on the y-axis. The transformed *p*-values have been re-scaled to range from 0 to 1 for visualisation, with darker colours representing greater statistical significance. Black outlines demarcate regions where TFCE *p*-values are below 0.05. Adolescence: *C3ar1*$^{+/+}$ *N* = 32 (15 males, 17 females); *C3ar1*$^{tm1Cge/tm1Cge}$ N = 32 (18 males, 14 females); Adulthood: *C3ar1*$^{+/+}$ *N* = 33 (16 males, 17 females) and *C3ar1*$^{tm1Cge/tm1Cge}$ *N* = 32 (17 males, 15 females).

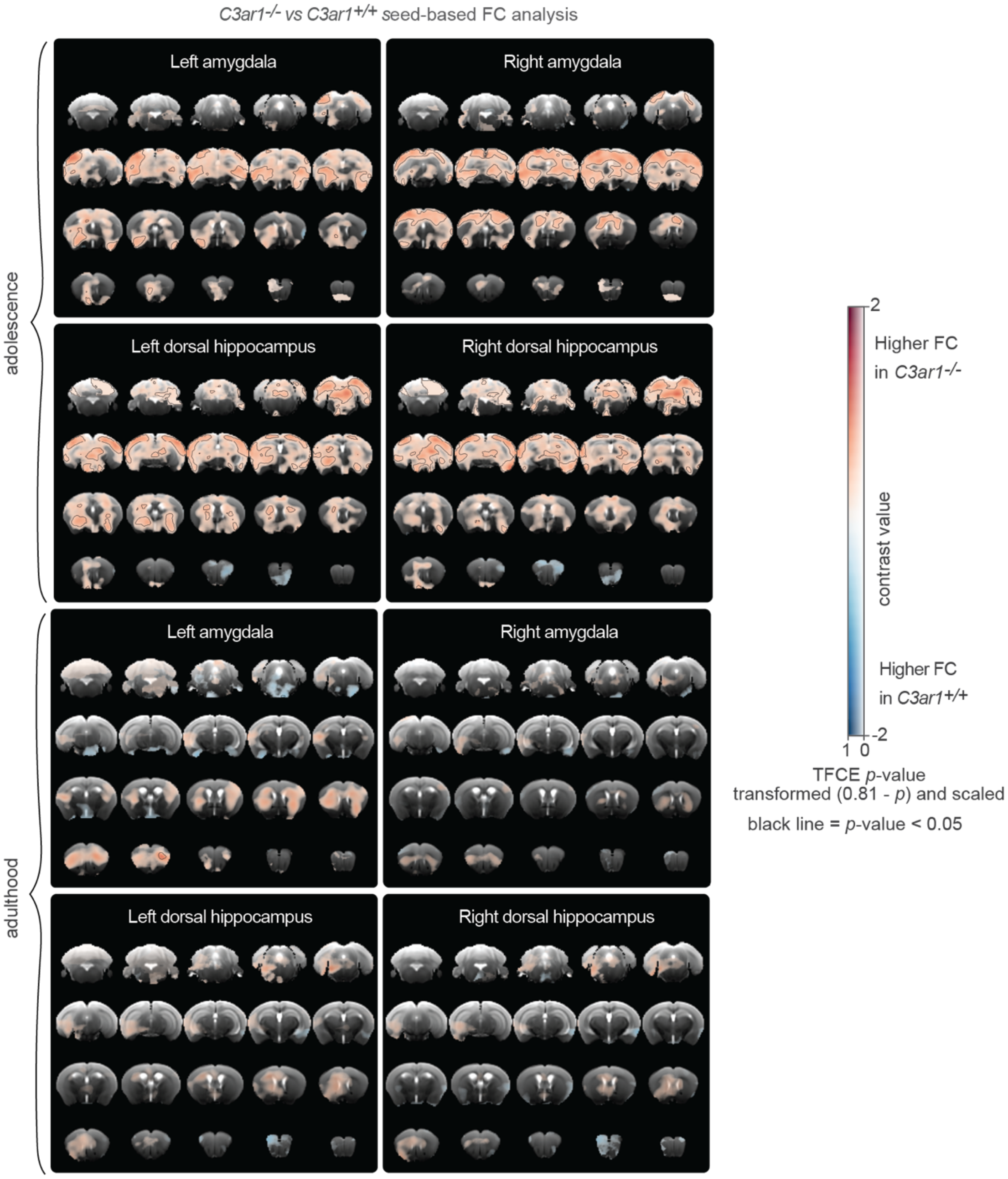

Supplemental figure 10 | Seed analysis of anxiety-related regions. Examples of seed-based FC maps showing voxel-wise group differences between *C3ar1*$^{tm1Cge/tm1Cge}$ and *C3ar1*$^{+/+}$ mice (using *t* tests) with seeds placed in bilateral dorsal hippocampus and amygdala in adolescence and adulthood datasets. The dual scale bar displays contrast value on the x-axis and threshold free cluster enhancement (TFCE) *p*-values (transformed 0.81–p) on the y-axis. Adolescence: *C3ar1*$^{+/+}$ *N* = 32 (15 males, 17 females); *C3ar1*$^{tm1Cge/tm1Cge}$ N = 32 (18 males, 14 females); Adulthood: *C3ar1*$^{+/+}$ *N* = 33 (16 males, 17 females) and *C3ar1*$^{tm1Cge/tm1Cge}$ *N* = 32 (17 males, 15 females).

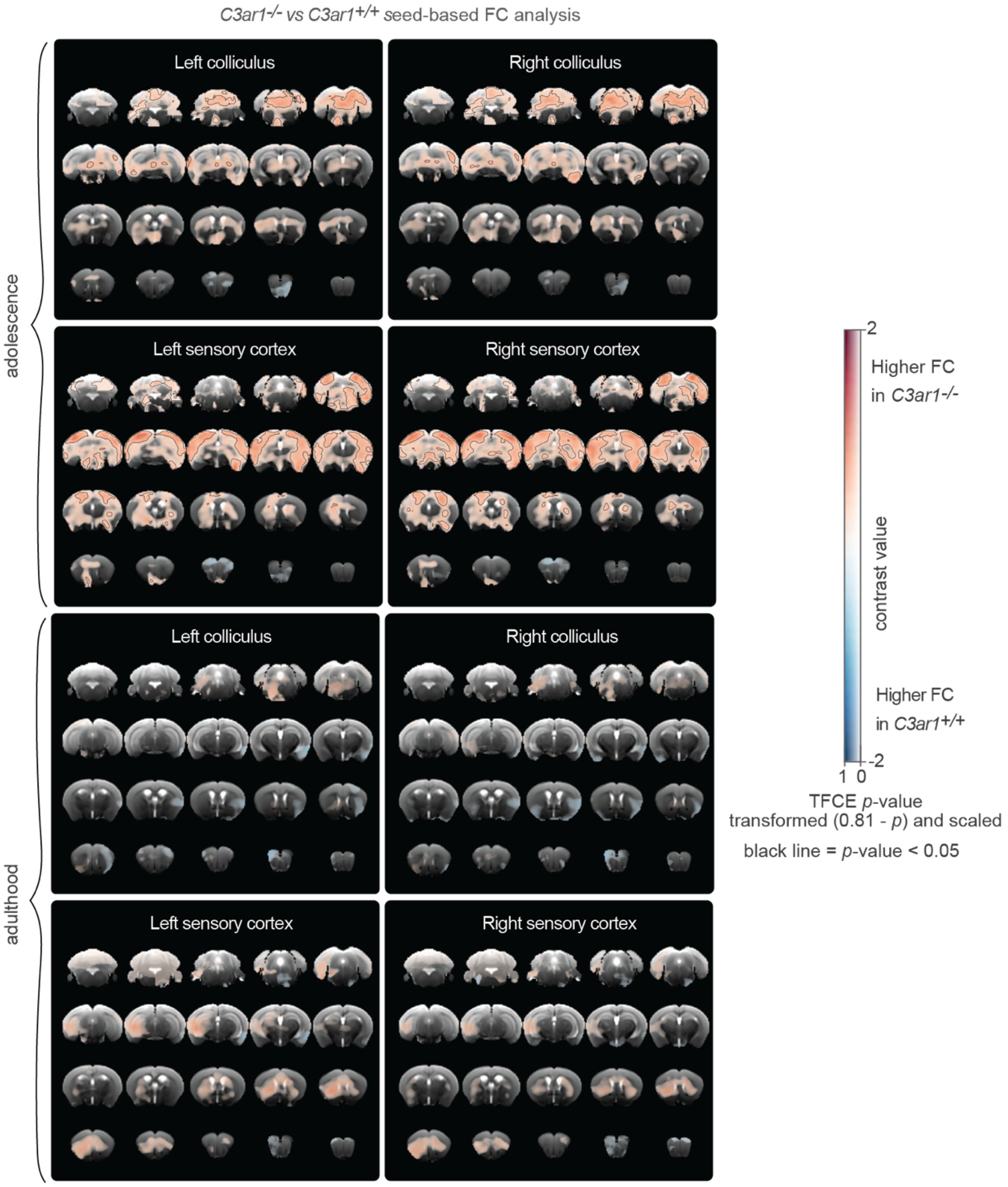

Supplemental figure 11 | Seed analysis of colliculus and sensory cortex connectivity. Seed-based functional connectivity (FC) maps showing voxel-wise group differences between *C3ar1*$^{tm1Cge/tm1Cge}$ and *C3ar1*$^{+/+}$ mice (using *t* tests) with seeds placed in bilateral colliculus and sensory in adolescence and adulthood datasets. The dual scale bar displays contrast value on the x-axis and threshold free cluster enhancement (TFCE) *p*-values (transformed 0.81−p) on the y-axis. Adolescence: *C3ar1*$^{+/+}$ $N = 32$ (15 males, 17 females); *C3ar1*$^{tm1Cge/tm1Cge}$ N = 32 (18 males, 14 females); Adulthood: *C3ar1*$^{+/+}$ $N = 33$ (16 males, 17 females) and *C3ar1*$^{tm1Cge/tm1Cge}$ $N = 32$ (17 males, 15 females).

Supplemental table 4 Anxiety-like behavioural outcome measures

Outcome measure	Comment
**Down with anxiety**	
OF duration core (30% of arena)	Commonly used metric to quantify anxiety-like behaviour. Similar measurements in *C3ar1* knockout mice were previously reported in 25% of arena,[6] 36% of arena[7] and 50% of arena,[8] with none finding significant differences compared to control. Open field centre duration is not always sensitive to anxiolytic or anxiogenic drugs.[9]
OF duration centre (70% of the arena)	Like the above but allows for more data to be gathered where exploration of the core 30% is insufficient.
EPM duration open	Increased with anxiolytics in rats[10] where it is regulated by the ventral hippocampus and the lateral septum.[11] Tested in *C3ar1* knockout mice twice, with one group noting increased anxiety-like behaviour[7] and another group not observing a difference.[8]
EPM duration middle	Rodents naturally seek enclosed spaces for protection. In the EPM tests, they first tend to remain in closed arms, then may pause in the middle section to assess safety before entering open areas. Higher anxiety would presumably be associated with more time spent also in this middle zone.
EPM bouts open	Like EPM duration open, also specifically validated in rats.[10]
EPM head dips	Anxiolytic drugs decrease head dip number and duration in the hole-board test in mice.[12] Tested in *C3ar1* mice[7] who saw decreased duration relative to wild-type.
**Up with anxiety**	
OF duration periphery	Thigmotaxis is a measure of anxiety-like behaviour in mice.[13]
OF latency to core 30%	Similar to core duration, mice who take longer to explore the centre can be more fearful.
OF latency to centre 70%	Similar to the above.
EPM latency to open arm	Tested in *C3ar1* knockout mice.[7] This group saw longer latency relative to wild-type.
EPM stretch attend postures	An increase in *C3ar1* knockout mice has been reported.[7]
EPM duration closed	Like thigmotaxis. An increase in *C3ar1* knockout mice has been reported.[7]

Supplemental table 5 Uncorrected *p* values for Cohort 1 behavioural measures shown in main text Figure 7.

Outcome measure	Test type	Genotype *F* stat	Genotype *p* value	Sex *F* stat	Sex *p* value	Interaction *F* stat	Interaction *p* value	*H* stat	4 groups *p* value
EPM bouts open	Kruskal							2.75	0.43
EPM head dips	Kruskal							1.37	0.71
EPM duration closed	ANOVA	0.38	0.54	0.16	0.69	2.85	0.10		
EPM duration middle	ANOVA	0.03	0.87	1.60	0.21	0.92	0.34		
EPM duration open	Kruskal							0.37	0.95
EPM latency middle	Kruskal							0.93	0.82
EPM latency open	Kruskal							1.94	0.59
EPM stretch attend postures	ANOVA	0.68	0.41	3.90	0.05	0.94	0.34		
OF distance centre	Kruskal							0.76	0.86
OF distance core	Kruskal							0.20	0.98
OF distance periphery	ANOVA	0.49	0.49	2.09	0.15	0.00	0.98		
OF duration centre	ANOVA	1.28	0.26	0.16	0.69	0.50	0.48		
OF duration core	Kruskal							0.55	0.91
OF duration periphery	ANOVA	1.28	0.26	0.17	0.69	0.50	0.48		
OF latency centre	Kruskal							0.33	0.95
OF latency core	Kruskal							1.97	0.58
OF loco speed	ANOVA	0.08	0.77	5.53	0.02	0.04	0.85		
OF loco total distance	ANOVA	0.02	0.89	3.30	0.07	0.00	0.96		
OF loco total duration	ANOVA	0.26	0.61	1.74	0.19	0.00	0.96		

Supplemental table 6 Uncorrected *p* values and effect sizes for Cohort 2 behavioural measures shown in main text Figure 7.

Outcome measure	*N* WT	*N* KO	Para-metric?	Test type	Test stat	*p* value	Effect size type	Effect size
ASR avg startle	19	20	FALSE	Kruskal	0.71	0.399	ε^2	-0.01
ASR baseline startle	19	20	FALSE	Kruskal	0.03	0.866	ε^2	-0.03
ASR habituation	19	20	TRUE	*t* test	-1.95	0.059	Cohen's *d*	-0.63
ASR latency	19	20	FALSE	Kruskal	0.97	0.325	ε^2	-0.001
ASR PPI global	19	20	TRUE	*t* test	1.42	0.163	Cohen's *d*	0.46
EPM bouts open	18	18	FALSE	Kruskal	0.41	0.523	ε^2	-0.02
EPM duration closed	18	18	TRUE	*t* test	0.44	0.662	Cohen's *d*	0.15
EPM duration middle	18	18	TRUE	*t* test	0.41	0.688	Cohen's *d*	0.14
EPM duration open	18	18	FALSE	Kruskal	0.84	0.359	ε^2	-0.001
EPM head dips	18	18	FALSE	Kruskal	0.01	0.987	ε^2	-0.03
EPM latency middle	18	18	FALSE	Kruskal	0.65	0.42	ε^2	-0.01
EPM latency open	18	18	FALSE	Kruskal	0.9	0.342	ε^2	-0.003
EPM stretch attend postures	18	18	TRUE	*t* test	1.08	0.288	Cohen's *d*	0.36
NOR test bouts familiar	19	20	FALSE	Kruskal	0.56	0.472	ε^2	-0.013
NOR test bouts novel	19	20	FALSE	Kruskal	0.17	0.683	ε^2	-0.023
NOR test familiar exploration	19	20	FALSE	Kruskal	0.11	0.736	ε^2	-0.024
NOR test latency novel	19	20	FALSE	Kruskal	1.74	0.187	ε^2	0.02
NOR test loco duration	19	20	TRUE	*t* test	1.14	0.261	Cohen's *d*	0.36
NOR test novel exploration	19	20	TRUE	*t* test	-0.6	0.523	Cohen's *d*	-0.21
NOR test RI	19	20	TRUE	*t* test	-0.42	0.673	Cohen's *d*	-0.14
NOR test total exploration	19	20	TRUE	*t* test	-0.55	0.584	Cohen's *d*	-0.18
NOR train bouts	19	20	TRUE	*t* test	0.5	0.618	Cohen's *d*	0.16
NOR train exploration duration	19	20	TRUE	*t* test	-0.20	0.840	Cohen's *d*	-0.07
NOR train locomotion duration	19	20	FALSE	Kruskal	2.94	0.087	ε^2	0.052
OF distance centre	19	20	TRUE	*t* test	2.88	0.007	Cohen's *d*	0.92
OF distance core	19	20	FALSE	*t* test	2.30	0.129	ε^2	0.04
OF distance periphery	18	20	TRUE	*t* test	1.78	0.084	Cohen's *d*	0.57
OF duration centre	19	20	FALSE	Kruskal	0.97	0.325	ε^2	-0.001
OF duration core	19	20	FALSE	Kruskal	1.78	0.182	ε^2	0.02
OF duration periphery	18	20	FALSE	Kruskal	0.62	0.43	ε^2	-0.01
OF latency centre	19	20	FALSE	Kruskal	0.001	0.978	ε^2	-0.03
OF latency core	19	20	FALSE	Kruskal	0.44	0.509	ε^2	-0.06
OF loco speed	19	20	TRUE	*t* test	1.33	0.190	Cohen's *d*	0.43
OF loco duration	19	20	TRUE	*t* test	1.78	0.083	Cohen's *d*	0.57
OF total distance	19	20	TRUE	*t* test	2.01	0.052	Cohen's *d*	0.64
PPI 12dB	19	20	TRUE	*t* test	0.28	0.783	Cohen's *d*	0.09
PPI 3dB	19	20	TRUE	*t* test	1.58	0.122	Cohen's *d*	0.51
PPI 6dB	19	20	TRUE	*t* test	1.99	0.054	Cohen's *d*	0.64

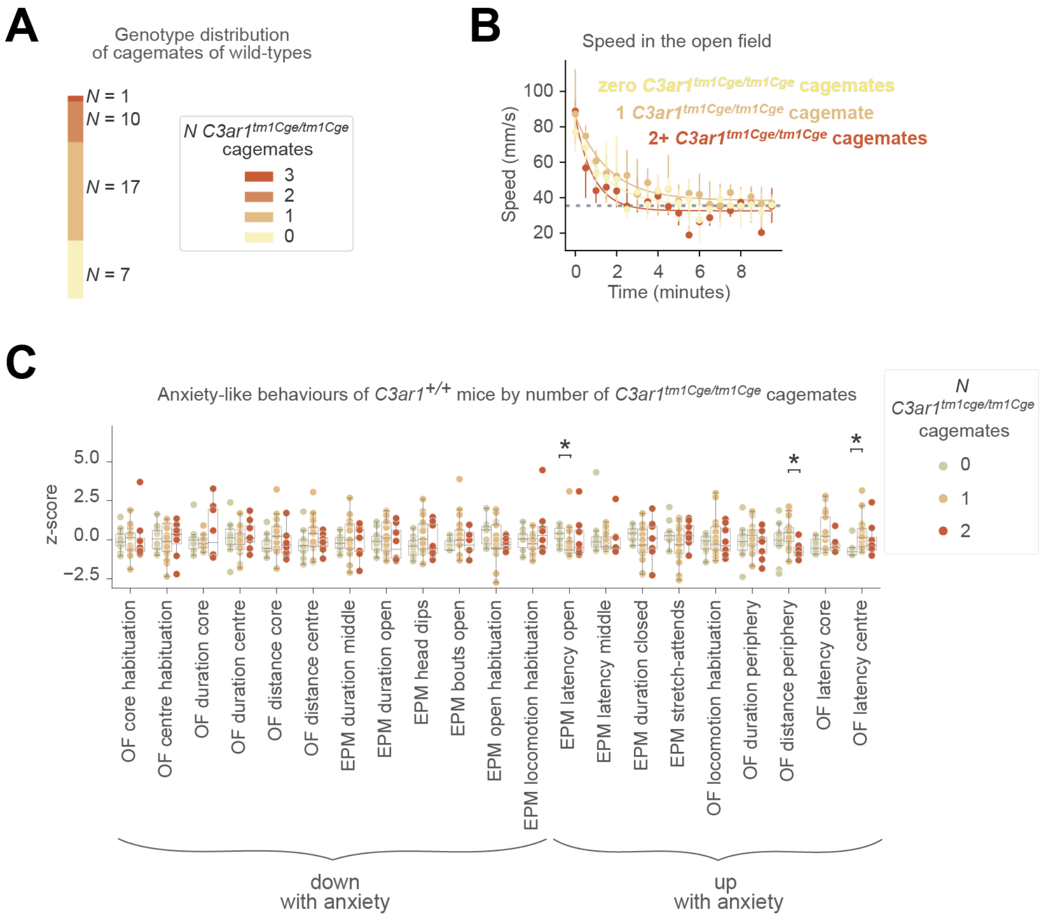

Supplemental figure 12 | Wild-type anxiety-like behaviour does not depend on the number of co-housed *C3ar1*-deficient mice. (**A**) 35 wild-type mice were housed with either zero, one, two or three *C3ar1^{tm1Cge/tm1Cge}* mice. (**B**) Wild-type speed in OF in the longitudinal study cohort separated by the number of *C3ar1^{tm1Cge/tm1Cge}* mice per cage. A two-way ANOVA was used to compare three experimental groups (0, 1, or 2–3 mutant cage-mates); post-hoc tests were not performed since only the effect of time was significant ($p < 0.001$). Data are presented as mean ± 95% CI. (**C**) Wild-type anxiety-like outcome measures separated by the number of *C3ar1^{tm1Cge/tm1Cge}* mice per cage (three groups; 0, 1 or 2–3 mutants). Kruskal Wallis tests followed by Dunn's test for multiple comparisons, not multiplicity corrected. * = p value < 0.05. (**B**–**C**) Each data point represents an individual mouse. Box plots show median, quartiles, and range. See (A) for the number of data points.

Supplemental references

1. Tournier JD, Smith R, Raffelt D, et al. MRtrix3: A fast, flexible and open software framework for medical image processing and visualisation. *Neuroimage*. 2019;202(116137):116137.

2. Avants BB, Tustison NJ, Song G, Cook PA, Klein A, Gee JC. A reproducible evaluation of ANTs similarity metric performance in brain image registration. *Neuroimage*. 2011;54(3):2033-2044.

3. Jenkinson M, Beckmann CF, Behrens TEJ, Woolrich MW, Smith SM. FSL. *Neuroimage*. 2012;62(2):782-790.

4. Smith SM, Jenkinson M, Woolrich MW, et al. Advances in functional and structural MR image analysis and implementation as FSL. *Neuroimage*. 2004;23 Suppl 1:S208-19.

5. Kim E, Carreira Figueiredo I, Simmons C, et al. Mapping acute neuroinflammation in vivo with diffusion-MRI in rats given a systemic lipopolysaccharide challenge. *Brain Behav Immun*. 2023;113:289-301.

6. Pozo-Rodrigálvarez A, Ollaranta R, Skoog J, Pekny M, Pekna M. Hyperactive Behavior and Altered Brain Morphology in Adult Complement C3a Receptor Deficient Mice. *Front Immunol*. 2021;12:406.

7. Westacott LJ, Humby T, Haan N, et al. Complement C3 and C3aR mediate different aspects of emotional behaviours; relevance to risk for psychiatric disorder. *Brain Behav Immun*. 2022;99:70-82.

8. Sun R, Tang MY, Yang D, et al. C3aR in the medial prefrontal cortex modulates the susceptibility to LPS-induced depressive-like behaviors through glutamatergic neuronal excitability. *Prog Neurobiol*. Published online April 17, 2024:102614.

9. Prut L, Belzung C. The open field as a paradigm to measure the effects of drugs on anxiety-like behaviors: a review. *Eur J Pharmacol*. 2003;463(1-3):3-33.

10. Pellow S, Chopin P, File SE, Briley M. Validation of open : closed arm entries in an elevated plus-maze as a measure of anxiety in the rat. *J Neurosci Methods*. 1985;14(3):149-167.

11. Trent NL, Menard JL. The ventral hippocampus and the lateral septum work in tandem to regulate rats' open-arm exploration in the elevated plus-maze. *Physiol Behav*. 2010;101(1):141-152.

12. Takeda H, Tsuji M, Matsumiya T. Changes in head-dipping behavior in the hole-board test reflect the anxiogenic and/or anxiolytic state in mice. *Eur J Pharmacol*. 1998;350(1):21-29.

13. Simon P, Dupuis R, Costentin J. Thigmotaxis as an index of anxiety in mice. Influence of dopaminergic transmissions. *Behav Brain Res*. 1994;61(1):59-64.
